# Financial Stress and Tobacco Expenditure in Australian Households: A Cross-Sectional Analysis of Prevalence and Association Across Wealth and Income Levels

**DOI:** 10.1093/ntr/ntaf102

**Published:** 2025-05-13

**Authors:** Koen Smit, Rowan Dowling, Robin Room, Anne-Marie Laslett, Ron Borland, Charles Livingstone, Heng Jiang

**Affiliations:** Centre for Alcohol Policy Research, La Trobe University, Victoria, Australia; Centre for Alcohol Policy Research, La Trobe University, Victoria, Australia; Centre for Alcohol Policy Research, La Trobe University, Victoria, Australia; Centre for Alcohol Policy Research, La Trobe University, Victoria, Australia; School of Psychology, Deakin University, Burwood, Victoria, Australia; School of Public Health and Preventive Medicine, Monash University, Victoria, Australia; Centre for Alcohol Policy Research, La Trobe University, Victoria, Australia; Melbourne School of Psychological Sciences, University of Melbourne, Victoria, Australia

## Abstract

**Introduction:**

Despite successful public health campaigns, tobacco use persists as a major cause of preventable illness and death. While tobacco taxation is recognized as an effective control strategy, concerns remain about potential financial strain on lower socioeconomic groups. This study investigates the relationship between household tobacco expenditure and financial stress in Australia, a country with high tobacco taxes and declining smoking rates.

**Methods:**

Household data from the 2015-16 Australian Household Expenditure Survey were analyzed (*N* = 10 036). Financial stress was measured using a scale based on nine self-reported indicators. Respondents were asked to report if their household had experienced any of these difficulties, for example, inability to pay utility bills or going without meals. Negative binomial regression models assessed the association between tobacco expenditure share and financial stress, adjusting for sociodemographic factors, household wealth, and other expenditures.

**Results:**

Financial stress was more prevalent among households that did (45.0%; (95% CI = 42.5 to 47.5)) versus did not (25.4%) purchase tobacco. All levels of tobacco expenditure were significantly associated with higher financial stress bivariably, after controlling for covariates. For instance, households in the second-lowest tobacco expenditure share quintile had a higher mean financial stress score than non-purchasing households (RR = 1.59, CI = 1.36 to 1.85, *p* < .001).

**Discussion:**

In Australia, financial stress is prevalent among tobacco-purchasing households, and household tobacco expenditure is significantly associated with increased financial stress even at modest levels of spending, that is, the lower quintiles of tobacco expenditure. These findings underscore the need for targeted policies to mitigate financial strain and support smoking cessation among vulnerable populations.

**Implications**

This study found that the prevalence of financial stress is higher in Australian households that purchase tobacco, regardless of their spending on tobacco. Although tobacco price increases reduce overall tobacco use, our study shows that increased prices exacerbate strain among financially disadvantaged smokers. Further research into associations between financial well-being and tobacco use is needed, both nationally and internationally. Longitudinal research should also examine the longer-term health and economic impacts mediated by financial stress.

## Introduction

Despite the accomplishments of public health campaigns in curbing tobacco use and deterring youth initiation, the consumption of tobacco remains a major cause of preventable illness and death in high-income countries.^1–3^ The World Health Organization advises that countries ensure that at least 75% of the final retail price of tobacco products is derived from taxation,^4^ and this practice has become more common in recent years, especially among high-income countries.^5^

In Australia, smoking prevalence has declined over two decades, with daily smoking rates decreasing from 24% in the early 1990s to about 12.2% in 2015-16.^1^ As overall smoking rates decline, there remains an important difference in smoking between lower and higher socioeconomic areas.^6–9^ In 2015-16, smoking rates among individuals in the lowest socioeconomic areas were approximately 2.7 times higher than those in the highest socioeconomic areas.^1^ It must be noted, however, that the highest decreases in smoking were found in the lowest socioeconomic areas between 2013 and 2016, that is, from 19.9% to 17.7%.

Lower socioeconomic status (SES) groups have been observed to be particularly responsive to tobacco price increases, likely due to financial constraints making smoking less available.^6^ For example, during the 1980s and early 1990s in the United Kingdom, low-income groups showed greater sensitivity to increases in cigarette prices.^2–4^ For this reason, tobacco taxes are often cited as an efficient policy option for addressing socioeconomic inequalities in smoking.^5^ However, relatively few studies have focused on the financial stress that tobacco expenditure might place upon people who continue to smoke despite price increases.

Financial stress has implications that extend beyond economic consequences, being closely associated with adverse health outcomes, particularly in mental health domains.^6,7^ Notably, while financial stress is linked to an increased risk of depression among adults, causality cannot be established due to the observational nature of most studies. However, evidence suggests that improvements in financial circumstances may rapidly mitigate these effects. This highlights the potential for prompt alleviation of financial stress to yield benefits within a relatively short period.

It remains unclear whether tobacco taxation could lead to undesirable consequences. Chaloupka and Warner’s influential review, published over two decades ago,^8^ was until recently the only comprehensive review of the outcomes of tobacco taxation. A recent review by DeCicca et al.^9^ concluded that tobacco taxation is broadly effective in reducing smoking prevalence. However, whether high tobacco expenditure could lead to poorer welfare was identified as an underexplored area. Moreover, the optimal level of taxation remained unclear, especially in relation to personal costs and the wider economic consequences of smoking.

Given the evidence linking SES negatively with tobacco dependence severity,^10,11^ there is a pressing need for further research on this topic. Although various psychosocial stressors have been studied in relation to SES and health, with mixed findings,^12^ stress was a significant predictor of both tobacco use and decreased tobacco cessation.^6,13–15^ Even though financial stress is closely related to economic circumstances, its connection to smoking remains under-researched.

Financial stress may play a role in explaining the socioeconomic gradient in tobacco use. Evidence from structural models suggests that, for older US adults, about a third of the association between SES and current smoking can be explained by the experience of chronic financial stress.^7^ Longitudinal analyses of the International Tobacco Control Four Country Survey, covering the United States, United Kingdom, Australia, and Canada,^16^ found that financially stressed smokers showed greater intention to cease use but were less likely to attempt to quit. Financially stressed smokers who did attempt to quit were more likely to resume smoking, indicating lower success rates in quitting than those who were not financially stressed. Hence, it is plausible that reduced tobacco affordability could, to some degree, reinforce smoking for financially stressed smokers who continue to smoke. Pathways which may explain this relationship are discussed by Mills et al.,^17^ who hypothesized that financial strain resulting from smoking can escalate stress directly or through housing instability, which in turn leads to increased smoking.

This study aims to address critical gaps in the literature by examining the relationship between household tobacco expenditure and financial stress in Australia, a country that has experienced dramatic changes in tobacco affordability and the social acceptability of smoking.^18^ The development of tobacco control policies that are both fair and effective requires good understanding of the complex relationship between tobacco affordability, financial stress, and tobacco use.^19^ By using recent data, this study provides valuable insights into the impact of increasing tobacco taxes on financial stress and smoking behavior in a setting characterized by low rates of tobacco use, high tobacco tax rate, and overrepresentation of disadvantaged groups among current smokers.^20^

Specifically, this study aims to investigate (1) the prevalence of financial stress among Australian tobacco-purchasing households. Moreover, we examine (2) the association between tobacco expenditure as a share of total expenditure and financial stress, including its correlation and magnitude. A secondary aim of this study was to estimate the prevalence of specific financial stress indicators among smokers.

## Methods

### Procedure

This study used data from the 2015-16 Household Expenditure Survey (HES)^21^ conducted by the Australian Bureau of Statistics. The survey adopted a stratified, multistage cluster sampling design and sampled from all households in Australian private dwellings except those in “Very Remote” areas (around 3% of total). The 2015-16 survey incorporated a supplementary sample of capital city households, which primarily sourced income from social welfare payments. Dwellings included permanent and nonpermanent (ie caravans, tents) structures on private property. A household was defined as one or more individuals in a dwelling sharing living essentials, excluding persons who primarily resided elsewhere. Households containing foreign diplomatic or military personnel were omitted. Of the 17 873 selected households, 14% (*n* = 2579) were uncontactable.

Each household received one “household” questionnaire, while members aged 15 and above were each given an “individual” questionnaire and expenditure diaries. One reference person per household was chosen, starting with the most selective (tenure) and then moving to less selective criteria until a single household reference person was identified (see “Household reference definition” in Supplementary Materials). Expenditure data were primarily collected from diary entries, with exceptions for large, sporadic expenses like whitegoods (eg refrigerator or stove), or regular outlays such as monthly bills, both collected within the household questionnaire. An individual’s diary data were deemed complete if at least one of their weekly diaries was valid.

Households were excluded by the Australian Bureau of Statistics if a principal income earner either lacked comprehensive diary data or omitted key questionnaire items like income details. For less critical items linked to a primary earner, or for any items related to other members, missing values were imputed by drawing from a demographically matched, randomly selected person. In total, 3638 households had at least one imputed value. The HES dataset contained the 10 046 households who agreed to participate and provided sufficient data. We analyzed data from 10 036 households, due to missing data on financial stress (*n* = 5), and households with questionable tobacco or alcohol expenditure (exceeding total service and goods expenditure, *n* = 5). The missingness pattern was assumed to be Missing at Random, as missing values likely depend on observed demographic characteristics. We conducted an additional sensitivity analysis to ensure that the imputation did not bias the results.

The study followed the STROBE guidelines (Strengthening the Reporting of Observational Studies in Epidemiology).^22^ Ethics approval for the study was obtained from the Human Ethics Sub-Committee of La Trobe University (No: HEC20076).

### Measures

#### Outcome Variables

The financial stress variable was based on seven core financial stress indicators^21^ (Could not pay for electricity, gas or telephone bills on time; Could not pay for car registration or insurance on time; Pawned or sold something; Went without meals; Unable to heat my home; Sought assistance from welfare/community organizations; Sought financial help from friends or family) and two additional financial stress indicators (If two thousand dollars can be obtained within a week; Spending more money than what is obtained), see supplements for details. With a score of 1 for each selected item, a financial stress score was created [range 0 to 9]. A modified parallel analysis was performed using 1000 bootstrapped samples, which is a statistical method used to determine the number of factors to retain in exploratory factor analysis. The bootstrapping approach ensures stability and reliability in the results by repeatedly resampling the data. The combination of statistical results (*p* = .004) and the scree plot (Figure S1) suggests strong statistical evidence against multidimensionality. This means that the financial stress items likely represent a single construct rather than multiple separate factors. This justified treating the sum of the items as a unidimensional scale^23^ showing satisfactory internal consistency^24^ with a Cronbach’s alpha of 0.73 (95% confidence interval [CI] = .71 to .74).

#### Exposure Variables

*Tobacco expenditure share* represented the percentage of household goods and services expenditure allocated to tobacco products. Households with tobacco expenditure were divided into five quantiles [range 1 (low) to 5 (high)] and an additional category [none] represented households with zero tobacco expenditure.

#### Covariates

Alcohol and gambling expenditures (over the last 4 weeks) were included as covariates and were represented with binary indicators [0 (no) and 1 (any)].

Equivalized household disposable income was calculated using the modified Organisation for Economic Co-operation and Development measure. This scales disposable income based on household composition by assigning points: 1 for the first adult aged 15 or older, 0.5 for each additional adult, and 0.3 for each child under 15. The equivalized household disposable income is determined by dividing the household’s disposable income by its total points, reflecting the income a single-adult household would need for a comparable standard of living. Households were then divided into five quantiles according to equivalized household disposable income.

Household wealth was described by liquidity ratio, the ratio of liquid assets (eg cash) to disposable income. Household wealth predicted financial stress independent of income in recent Australian evidence,^25^ possibly due to providing a buffer against financial stresses in the short term.^26^

Other sociodemographic covariates included housing tenure (owned/mortgaged/rented/other), location within a state capital city’s greater metropolitan region (yes/no), lone parent with dependent children (yes/no), and three characteristics of the reference person: gender (Male/Female), age (15 to 34 years, 35 to 54 years, 55 years or over), and educational attainment (up to year 12, certificate to diploma, bachelor degree or higher).

### Statistical Analysis

To assess prevalence of financial stress, we calculated the median share of total goods and services expenditure allocated to tobacco products for the subgroup of respondents with any level of tobacco expenditure. Crosstabs were used to estimate financial stress prevalence and total scores by household tobacco status. Differences were examined with the Rao-Scott adjusted chi-square tests. For population prevalence and household counts, we applied jackknife replicate weights, which were provided by the Australian Bureau of Statistics and employed for the computation of standard errors.^27,28^ Confidence intervals were calculated with the Korn-Graubard method, chosen for its accuracy for proportions nearing 0 or 1.^29^

In addition, to estimate the prevalence of specific financial stress indicators among tobacco-purchasing households, we calculated the weighted prevalences of each indicator and compared it with non-tobacco-purchasing households.

#### Regression Analysis

To examine associations between tobacco expenditure and financial stress, regression analyses using count variable models were used.^30^ To assess the robustness of the association, we ran three multivariable models, as well as a bivariable. In the first multivariable model, we adjusted for demographic characteristics, including age and gender of the reference person. In a second model, we further adjusted for household characteristics including wealth, house tenure, household composition, and location. In the third model, we included additional covariates for household expenditure on alcohol and gambling.

As outcome data were anticipated to be over-dispersed due to intercorrelation, exceeding the capacity of a Poisson distribution, a negative binomial distribution was used. Financial stress count was modeled via a negative binomial Generalized Linear Model using a log link function and fit using Maximum Likelihood Estimation. As the link function complicated the direct interpretation of the findings, coefficients in our model were converted to a ratio through exponentiation to provide interpretable results. The exponentiated coefficients represent relative change in outcome for a one-unit shift in continuous variables or versus a categorical variable’s reference level. However, instead of predicting the odds of an event across conditions, our model predicted the outcome’s mean value across conditions. Throughout this article, we refer to these exponentiated coefficients as rate ratios.

### Model Fit and Sensitivity Analyses

The Generalized Variance Inflation Factor was used to examine multicollinearity, and a threshold of ≥2 was indicative of multicollinearity, analogous to a Variance Inflation Factor of 4 (37). Sensitivity analyses were conducted for alternative specifications of the outcome variable (see Supplementary Information). The three logistic regressions used the following outcomes: (1) any financial stress out of the nine items (ie ≥ 1 financial stress vs. 0), (2) any financial stress out of seven core items (ie ≥ 1 financial stress vs. 0), and (3) predicting the “went without meals” item (yes vs. no). A fourth sensitivity analysis investigated whether our main findings were sensitive to the imputation method used by the Australian Bureau of Statistics, by excluding imputed cases. All statistical analyses were conducted using R Statistical language (version 4.2.2; R Core Team, 2022), and example R code is available in the Supplementary Materials. Analyses were primarily conducted using the packages *survey*^31^ for descriptive analyses, and *glmmTMB*^32^ for regression analyses.

## Results

### Respondent Characteristics

Table 1 shows unweighted respondent characteristics and weighted percentages. The study sample included 10 036 households containing 23 508 individuals. Most reference persons were male (59%) and were under 55 years old (60%). Most households (63%) were in a metropolitan area and reported no tobacco purchasing (82%). Lower equivalized household disposable income levels and high tobacco expenditure appear to be overrepresented in the sample, likely due to the oversampling of city-dwelling welfare recipient households, who were typically older and had lower incomes. Considering only the tobacco-purchasing households, the median share of total goods and services expenditure allocated to tobacco products was 5.34% (25th percentile = 2.50%, 75th percentile = 10.05%) (not shown in table).

**Table 1.** Tobacco expenditure and other characteristics of the 2015-16 Australian Household Expenditure Survey sample

	*n*, unweighted	%, unweighted	%, weighted
Total sample	10 036	100%	100%
Tobacco expenditure quintile^a^			
None ($0)	8360	83.3%	82.0%
1 (Lowest)	296	3.0%	3.5%
2	302	3.0%	3.5%
3	327	3.3%	3.5%
4	343	3.4%	3.5%
5 (Highest)	408	4.1%	3.5%
Equiv. disposable income^b^ quintile			
5 (Highest)	1729	17.2%	20.2%
4	1774	17.7%	19.6%
3	1829	18.2%	19.1%
2	2211	22.0%	19.2%
1 (Lowest)	2493	24.8%	20.0%
Gender^c^			
Male	5631	56.1%	59%
Female	4405	43.9%	41%
Age^c^			
15-34	1735	17.3%	21.6%
35-54	3513	35.0%	38.4%
55+	4788	47.7%	40.0%
Liquidity			
Low	3438	34.3%	33.3%
Middle	3179	31.7%	33.4%
High	3419	34.1%	33.3%
Tenure			
Owned outright	3568	35.6%	30.2%
Mortgaged	3271	32.6%	37.3%
Rented	2973	29.6%	30.2%
Other	224	2.2%	2.3%
Household location			
Greater capital city	7429	74.0%	63.2%
Rest of state	2607	26.0%	36.8%
Education			
Bachelor degree or higher	2538	25.3%	28.2%
Certificate to diploma	3658	36.5%	37.0%
No post-secondary qualifications	3840	38.3%	34.8%
Lone parent			
No	9087	90.5%	92.0%
Yes	949	9.5%	8.4.0%
Gambling (any expenditure)			
No	7222	72.0%	72.7%
Yes	2814	28.0%	27.3%
Alcohol (any expenditure)			
Yes	5138	51.2%	55.0%
No	4898	48.8%	45.0%

^a^Tobacco expenditure levels = either zero expenditure or into five equally distributed household expenditure quintiles.

^b^Disposable income = gross income minus income tax and levies, that is, the net income available for consumption and saving.

^c^Gender and age are the characteristics of the (randomly selected) household referenced person who was the principal income earner (please see more information in supplements).

### Prevalence

**Figure 1.**
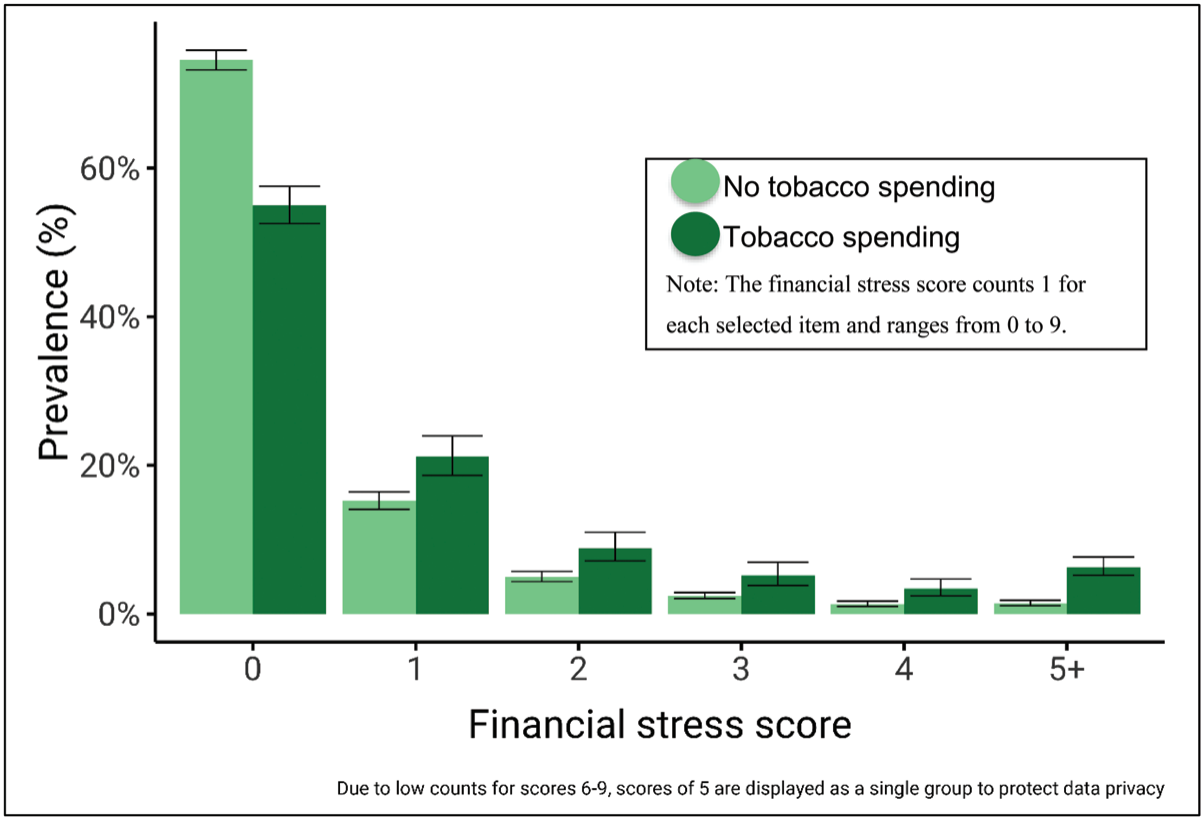
Prevalence of financial stress (count) indicators by household tobacco status.

Figure 1 presents survey-weighted prevalence estimates of financial stress count by tobacco status. There were an estimated 1.57 (95% CI = 1.47 to 1.67) million Australian households with tobacco expenditure in 2015-16. Most tobacco-purchasing households reported no financial stress indicators (55.0%; 95% CI = 52.5 to 57.5), but an estimated 45.0% (95% CI = 42.5 to 47.5) reported one or more indicators of financial stress. In contrast, the remaining 7.39 (95% CI = 7.29 to 7.48) million households without tobacco expenditure were comprised of an estimated 74.6% (95% CI = 73.2 to 75.9) households with no financial stress indicators and 25.4% (95% CI = 24.1 to 26.8) households with one or more financial stress indicators. The difference in the distribution of financial stress scores between households with and without tobacco expenditure was statistically significant (Rao & Scott adjusted Pearson’s χ^2^(9): 385.0, *p* < .001), as represented by the non-overlapping confidence intervals in Figure 1.

Table 2 shows survey-weighted prevalence estimates for individual financial stress items, both overall and by household tobacco status. There was evidence (*p* < .001) that each of the nine financial stress items were individually more prevalent among households with tobacco expenditure than those without. Most items were approximately twice as prevalent in households with tobacco expenditure, except for “Household typically spends more money than it gets,” which appeared to be less strongly correlated with tobacco expenditure.

**Table 2.** Prevalence of distinct financial stress indicators: overall and in households with and without tobacco expenditure

Financial stress indicators	Overall sample (*n* = 10 036) % (95% CI)	Household tobacco expenditure		
		No, % (95% CI)	Yes, % (95% CI)	χ² *p*-value
Could not pay bills on time	9.66 (8.97, 10.4)	7.74 (6.98, 8.58)	18.7 (16.3, 21.4)	<.001
Could not pay car registration or insurance on time	3.95 (3.48, 4.47)	2.94 (2.49, 3.47)	8.67 (7.18, 10.4)	<.001
Pawned or sold something	2.48 (2.04, 3.01)	1.79 (1.39, 2.29)	5.73 (4.38, 7.45)	<.001
Went without meals	2.73 (2.32, 3.20)	1.98 (1.55, 2.53)	6.23 (4.98, 7.76)	<.001
Unable to heat home	2.26 (1.89, 2.70)	1.75 (1.38, 2.21)	4.67 (3.61, 6.02)	<.001
Sought assistance from welfare/community organization	2.54 (2.12, 3.05)	1.61 (1.25, 2.07)	6.93 (5.42, 8.83)	<.001
Sought financial help from friends or family	7.03 (6.47, 7.64)	5.88 (5.30, 6.51)	12.5 (10.8, 14.3)	<.001
Household spend more money than they get weekly	12.8 (12.0, 13.7)	11.8 (11.0, 12.7)	17.4 (15.2, 19.9)	<.001
Not able to raise $2000 for something important within a week	13.2 (12.3, 14.1)	10.8 (9.82, 11.8)	24.8 (22.5, 27.3)	<.001

Pearson’s χ²: Rao and Scott adjustment.

CI = confidence interval.

#### Model Fit and Sensitivity

We found no evidence of multicollinearity between the predictor variables. Data were over-dispersed relative to the Poisson model (*p* < .001), confirming our choice of a negative binomial model.

Several sensitivity analyses were conducted to assess the robustness of our findings, the results of which can be found in the Supplementary Materials. Alternative models using logistic regression with different definitions of financial stress yielded similar results. Additionally, replicating the main analysis using only complete cases (excluding imputed data) did not substantially alter the observed relationship. In all alternative models, any tobacco expenditure remained a statistically significant predictor of a similar magnitude to that found in the main regression.

#### Regression

The multiple regression analyses considered included three models. Model 1 predicted financial stress score (counted 1-9) using tobacco expenditure, income, and the gender and age of the household reference person. All predictors were statistically significant (*p* < .001). Households with the lowest quintile of tobacco expenditure had a 76% higher financial stress score than households without tobacco expenditure. Households with the second-lowest quintile tobacco expenditure had a 122% higher financial stress score than households without tobacco expenditure. The degree of elevation of the financial stress score did not vary substantially between the four higher-quintile (second to fifth) stress scores.

Model 2 added liquidity, household tenure, location, lone parent household, and household reference person educational attainment as predictors. All predictors were statistically significant, indicating that women, higher age (except 35-54 years), those with less liquidity, paying mortgage or rent, being in a capital city, lower education, and being a lone parent reported more financial stress. Tobacco expenditure and financial stress score were significantly associated in this model. Estimates ranged from an increase in financial stress of 41% for the highest quintile tobacco expenditure to an increase of 57% for the second-lowest quintile.

Model 3 further included gambling participation and alcohol expenditure in the model. The observed relationship of tobacco expenditure and financial stress and other predictors persisted, except for household location. Neither gambling nor alcohol, at the low level of involvement tested, were statistically significant predictors of financial stress. Estimates of the association of tobacco expenditure and financial stress remained almost identical to the previous model. The results from the bivariable models confirmed correlations between various predictors and financial stress, consistent with the findings from the multivariable models. They revealed that a higher share of tobacco expenditure is associated with a greater likelihood of experiencing financial stress.

## Discussion

The present study explored the cross-sectional association between tobacco expenditure and financial stress in Australian households between July 2015 and June 2016. The results revealed a striking disparity in the prevalence of financial stress indicators between households with and without tobacco expenditure. All nine indicators demonstrated significantly higher prevalence among households purchasing tobacco, often by several-fold. For example, 6% of tobacco households reported forgoing meals compared to 2% of non-purchasing households. Similarly, 5% of tobacco households were unable to afford heating, compared to 2% of non-purchasing households. These differences highlight the disproportionate financial stress experienced by households that purchase tobacco.

Importantly, tobacco expenditure consistently emerged as a significant predictor of elevated financial stress scores, independent of the level of spending. Notably, in the bivariable model, the magnitude of financial stress consistently increased with higher proportions of expenditure allocated to tobacco; however, this pattern was inconsistent, highlighting the need for cautious interpretation (Table 3). Moreover, this result was absent in the multivariable models once covariates such as income, wealth, and other expenditures were accounted for. Despite these confounding factors, tobacco expenditure remained a significant predictor of financial stress, suggesting an independent contribution to financial stress.

**Table 3.** Models assessing the relationship between tobacco expenditure and financial stress score (count), adjusting for sociodemographic and other household factors

	Multivariable (1)			Multivariable (2)			Multivariable (3)			Bivariable (4)		
Characteristic	RR	95% CI	*p*-value	RR	95% CI	*p*-value	RR	95% CI	*p*-value	RR	95% CI	*p*-value
Tobacco expenditure^a^												
None (ref)	—	—		—	—		—	—		—	—	
1 (Lowest)	1.76	1.45, 2.13	<.001	1.43	1.20, 1.69	<.001	1.45	1.22, 1.72	<.001	1.69	1.37, 2.08	<.001
2	2.22	1.85, 2.66	<.001	1.57	1.34, 1.84	<.001	1.59	1.36, 1.86	<.001	2.43	1.99, 2.96	<.001
3	1.90	1.58, 2.27	<.001	1.44	1.23, 1.69	<.001	1.46	1.25, 1.71	<.001	2.22	1.82, 2.71	<.001
4	2.19	1.84, 2.62	<.001	1.54	1.32, 1.79	<.001	1.56	1.34, 1.81	<.001	2.57	2.11, 3.13	<.001
5 (Highest)	2.09	1.75, 2.49	<.001	1.41	1.21, 1.65	<.001	1.43	1.23, 1.68	<.001	2.50	2.05, 3.04	<.001
Equiv. disposable income^b^												
5 (Highest) (ref)	—	—		—	—		—	—		—	—	
4	2.26	1.93, 2.65	<.001	1.85	1.59, 2.15	<.001	1.84	1.58, 2.15	<.001	2.37	2.02, 2.79	<.001
3	3.57	3.06, 4.16	<.001	2.80	2.42, 3.25	<.001	2.79	2.40, 3.23	<.001	3.75	3.21, 4.39	<.001
2	5.08	4.36, 5.91	<.001	3.40	2.93, 3.94	<.001	3.36	2.89, 3.89	<.001	4.90	4.20, 5.71	<.001
1 (Lowest)	7.79	6.70, 9.05	<.001	5.19	4.48, 6.01	<.001	5.09	4.38, 5.91	<.001	7.00	6.02, 8.14	<.001
Gender^c^												
Male (ref)	—	—		—	—		—	—		—	—	
Female	1.24	1.14, 1.34	<.001	1.14	1.06, 1.23	<.001	1.14	1.06, 1.23	<.001	1.44	1.32, 1.57	<.001
Age^c^												
15-34 (ref)	—	—		—	—		—	—		—	—	
35-54	0.87	0.79, 0.96	.006	1.02	0.94, 1.12	.6	1.03	0.94, 1.12	.6	0.92	0.82, 1.02	.10
55+	0.41	0.37, 0.45	<.001	0.86	0.77, 0.96	.007	0.86	0.78, 0.96	.009	0.51	0.45, 0.57	<.001
Liquidity												
Low (ref)				—	—		—	—		—	—	
Middle				0.38	0.35, 0.41	<.001	0.38	0.35, 0.41	<.001	0.25	0.23, 0.28	<.001
High				0.26	0.24, 0.29	<.001	0.26	0.24, 0.29	<.001	0.15	0.13, 0.17	<.001
Tenure												
Owned outright (ref)				—	—		—	—		—	—	
Mortgaged				1.74	1.54, 1.96	<.001	1.74	1.54, 1.96	<.001	2.03	1.81, 2.27	<.001
Rented				2.37	2.10, 2.67	<.001	2.35	2.09, 2.65	<.001	4.96	4.44, 5.53	<.001
Other				1.98	1.57, 2.50	<.001	1.97	1.56, 2.49	<.001	2.99	2.31, 3.87	<.001
Household location												
Greater capital city (ref)				—	—		—	—		—	—	
Rest of state				0.93	0.86, 1.00	.044	0.93	0.87, 1.00	.057	1.17	1.07, 1.27	<.001
Education^c^												
Bachelor degree or higher (ref)				—	—		—	—		—	—	
Certificate to diploma				1.20	1.08, 1.32	<.001	1.20	1.09, 1.33	<.001	1.82	1.63, 2.03	<.001
No post-secondary qualifications				1.16	1.05, 1.29	.004	1.17	1.05, 1.29	.003	2.23	2.00, 2.49	<.001
Lone parent												
No (ref)				—	—		—	—		—	—	
Yes				1.48	1.33, 1.65	<.001	1.47	1.32, 1.64	<.001	2.74	2.41, 3.12	<.001
Gambling (any expenditure)												
No (ref)							—	—		—	—	
Yes							0.95	0.87, 1.04	.3	0.74	0.67, 0.81	<.001
Alcohol (any expenditure)												
Yes							—	—		—	—	
No							1.04	0.96, 1.12	.3	1.52	1.40, 1.65	<.001

^a^Tobacco expenditure levels = either zero expenditure or into five equally distributed household expenditure quintiles.

^b^Disposable income = gross income minus income tax and levies, that is, the net income available for consumption and saving.

^c^Gender, age and education are the characteristics of the household referenced person, who is a person chosen to represent a household in a survey (please see more information in supplements).

CI = confidence interval; RR = risk ratio.

The findings of this study are mainly in line with previous literature, which consistently highlights the financial burden of tobacco use on low-income households and its potential role in perpetuating socioeconomic inequalities.^10,33^ Several potential pathways may explain the relationship between tobacco expenditure and financial stress. The indirect costs of tobacco use, such as health care expenses from smoking-related illnesses and lost productivity, likely exacerbate financial stress over the long term by decreasing household resources.^8^ However, many smoking-related diseases take decades to manifest,^34^ suggesting that more immediate stressors may play a stronger role in the short term. The persistence of financial stress disparities between tobacco and non-tobacco households, even after adjusting for age, indicates that immediate factors also play a role. Spending on tobacco products may drain household resources, limiting funds available for essential needs like food, housing, and health care. This can create a stressful environment that may encourage the continuation of smoking, despite its financial drawbacks.^16^ Furthermore, tobacco use is associated with serious health problems, such as respiratory illnesses, heart disease, and cancer. These conditions often lead to increased medical expenses, which can exacerbate financial difficulties, particularly for those without adequate health insurance. Tobacco use may lead to absenteeism from work due to illness, lower work performance, or even job loss in extreme cases, reducing income and exacerbating financial stress.

Financial stress may also contribute to intergenerational patterns of smoking. Children in these families may be at higher risk of taking up smoking themselves. For example, through exposure to parental smoking^35^ or indirectly through chronic financial stress that reduces the capacity to support cessation efforts.^7^ In addition, psychological stress, limited social support, barriers to accessing health care, and reduced self-efficacy have been identified as predictors of smoking initiation and continuation.^33,36^ These could further strain financial stability and exacerbate the cycle of tobacco use and economic hardship.

Research suggests that wealth acts as a buffer against financial shocks in the short term,^26^ enabling households to utilize existing resources during economic challenges and thereby mitigate their impact. Additionally, wealth can reflect a historical accumulation of income and serve as a proxy of long-term SES,^25^ which is particularly significant given the intergenerational patterns of smoking behaviors shaped by familial socioeconomic circumstances.^35,37^ Moreover, wealth is potentially indicative of financial literacy, a factor that has shown a positive correlation with financial well-being in prior studies.^38^ For these reasons, potential associations between wealth and financial literacy, tobacco expenditure, and health outcomes warrant further investigation.

A significant dose-response relationship was found between alcohol expenditure share and financial stress in our previous study.^39^ Thus, we expected that higher tobacco expenditure share may correlate with increased financial stress. However, we found no evidence for a dose-response relationship between tobacco expenditure share and financial stress; most of the difference was observed between no expenditure and any expenditure on tobacco (Table 3). This prompts consideration of other influencing factors beyond the immediate effects of tobacco expenditure. For instance, individuals from financially stressed households may use tobacco as a coping mechanism for emotional stress, as nicotine offers a temporary calming or stimulating effect.^6^ Moreover, previous research has found that even during times of financial hardship, financial stress may drive individuals to smoke more.^40^ These patterns may significantly influence those from tobacco-purchasing households, especially those who already experience socioenvironmental stress such as unemployment, family disruptions, or housing instability. This may exacerbate financial difficulties^41^ and lead to increased psychological stress. Financially stressed individuals may lack access to resources like counseling or stress management programs,^42^ making tobacco one of the few accessible ways to manage their emotional challenges. Future studies should examine whether personal traits such as impulsivity or a propensity for risk-taking behavior affect both financial decisions and tobacco consumption patterns.

## Limitations

While our study provides valuable insights into the relationship between tobacco expenditure and financial stress, several limitations should be acknowledged. The cross-sectional design provides little evidence on causality. Moreover, the potential remains for further confounding factors to be involved, for example, geographic variations in exposure to tobacco marketing, social norms surrounding smoking, and neighborhood-level socioeconomic characteristics. Additionally, psychological factors such as individual stress propensity or sense of control over financial circumstances could contribute to the observed association.

Second, while our statistical model accounted for the accumulation of financial stress indicators, it assigned equal weight to each indicator. Financial strains such as food insecurity are considered to represent a more severe level of deprivation compared to others. Consequently, future research could explore alternative analytical approaches to account for the varying severity of financial stress indicators.

Lastly, the conclusions from this study are based on data from 2015 to 16. Due to the impact of the coronavirus disease of 2019 (COVID-19), the 2021-22 Australia household expenditure survey was suspended and eventually not released. Therefore, the Household Expenditure Survey 2015-16 is the most recent available data for such analysis in Australia. This needs to be considered when interpreting the current findings. Furthermore, caution should be exercised when generalizing current results to other countries, particularly where cigarette prices are lower. In these contexts, the financial burden of tobacco expenditure may differ, potentially changing the association between tobacco expenditure and financial stress.

## Implications

### Future Research

To further understanding of the mechanisms behind the observed link between tobacco use and financial stress, future research should explore the nuances of household expenditure patterns by comparing spending habits between smoking and nonsmoking households across various income and wealth levels. Analyzing how households distribute their budget across essential and discretionary expenditure categories, such as housing, food, transportation, and leisure, might reveal whether tobacco consumption compromises essential needs or necessitates reductions in other spending areas. Second, to understand the broader implications of financial stress, further studies should examine the consequences of financial stress among smoking households, particularly its predictive value for long-term health outcomes and material deprivation. In addition, qualitative studies are warranted to explore how households that purchase tobacco perceive and respond to financial stress, providing insight into the coping strategies they employ. Third, there is a need for more data to properly investigate the complex relationships of socioeconomic factors, financial stress, and smoking behaviors.^11^ Despite extensive global data from the World Health Organization’s Global Tobacco Epidemic reports across many domains, these do not currently report within-country socioeconomic disparities in smoking prevalence.^43^ Addressing this gap could yield vital information on global tobacco-related inequities and help tailor more effective interventions. Given Australia’s position with high cigarette prices and stringent tobacco control measures, it presents a compelling case study for investigating how socioeconomic factors influence tobacco affordability and smoking behaviors. If tobacco use is contributing to financial stress, it also suggests that this should be taken into consideration when raising taxes on these products, as it has the potential to exacerbate the problem.

### Policy Implications

This study’s findings suggest that policies aimed at reducing tobacco affordability and accessibility need to consider their impact on financial stress in tobacco-using households. Importantly, our study demonstrates that any tobacco expenditure was associated with higher financial stress, and there was little variation in stress by the amount of tobacco expenditure. This may imply that price increases could have a uniform effect among different levels of smoking. Taken together with preliminary evidence suggesting that financial stress could hinder a smoker’s cessation efforts,^16^ our findings raise concerns that raising tobacco prices might exacerbate strain among financially disadvantaged smokers, thereby decreasing their ability to quit.

There are substantial opportunities which are often overlooked in favor of pricing strategies.^44^ One potential strategy to get people out of this negative loop is the MPOWER framework, designed by the WHO. This framework includes raising taxes as one component among several tobacco control strategies. It also emphasizes strategies to monitor tobacco use, protect from exposure, offer help to quit, warn about smoking harms, and enforce bans.

Other examples are strategies targeting supply, including increased regulation of tobacco sales and a reduction in the number of retail outlets selling tobacco products. Many antismoking measures have intentionally sought to make smoking socially undesirable with the result that the social acceptability of tobacco smoking has substantially changed across many high-income countries.^18,45^ While stigmatizing smoking may promote public health aims, it also carries the risk of marginalizing vulnerable groups and even reducing their engagement with cessation support.^46^ By understanding the social and identity-related dimensions of smoking within these communities,^47^ and by examining the potential of supply-side interventions, researchers could develop more effective tobacco control strategies. Altogether, future tobacco policies should seek a balance between reducing tobacco consumption through pricing and accessibility measures and minimizing unintended negative consequences such as financial stress and social marginalization.

## Conclusion

Our findings suggest that, while tobacco-purchasing households reported almost twice the number of financial stress indicators compared to non-purchasing households, the proportion of total expenditure spent on tobacco products did not appear to drive financial stress for most tobacco-purchasing households. This work contributes to the growing body of evidence regarding financial hardship and tobacco use, particularly among low-income households. Further research will be required to better understand how tobacco and financial stress are related, especially studies that are well-controlled for both economic and psychological factors.

## Supplementary material

Supplementary material is available at *Nicotine and Tobacco Research* online.

ntaf102_suppl_Supplementary_Data

ntaf102_suppl_Supplementary_Materials

## Data Availability

The summary and brief of Australian Household Expenditure Survey data were published on the ABS web site, and project data are available to the project investigators only (Please see https://www.abs.gov.au/statistics/economy/finance/household-expenditure-survey-australia-summary-results).
