# Supplementary material for: Financial Stress and Tobacco Expenditure in Australian Households: A Cross-Sectional Analysis of Prevalence and Association Across Wealth and Income Levels
Source: Nicotine Tob Res. 2025 May 13;28(1):117–27. doi: 10.1093/ntr/ntaf102 (PMC12723215; doi:10.1093/ntr/ntaf102)
Supplement: ntaf102_suppl_Supplementary_Data [file ntaf102_suppl_supplementary_data.docx]

---

title: "HES_Handover"

---

## Setup

#### Font

#This code block configures the fonts which are used in the graph output later.

```{r font_setup}

#| include: false

#library("Cairo") # platform-independent graphics device

#knitr::opts_chunk$set(dev = "CairoPNG")

library(showtext)

font_add_google("Roboto", "Roboto")

```

#### Data import

This code block calls some of my scripts (found in the 'scripts/' directory). These scripts first import the data from the research drive and then does some preliminary operations to the variables to ready them for analysis.

```{r data_read}

#| include: false

#| cache: true

#| cache-vars: household

library(fs)

library(here)

source(set_here())

source(here("scripts/01setup.R"))

source(here("scripts/02import_and_clean.R"))

```

#### Packages import

This code block imports the packages that will be used in the rest of the code.

```{r imports}

#| include: false

#| echo: false

set.seed(4567)

library(tab) # Then load it

# Other imports

library(survey) # Deal with survey data

library(srvyr) # Deal with survey data in a 'tidyverse'-friendly manner

#library(svydiags) # Regression diagnostics for survey methods

library(flextable) # Output table builder

library(ftExtra) # Extra functions for flextable

library(broom.helpers)

library(gt) # Another table-building package

library(gtsummary) # Create publication-ready tables, uses gt as backend

library("RColorBrewer") # Load RColorBrewer

library(ltm) # used for Item Response analyses.

library(glue) ## allows easy manipulation of strings

library(tidyverse) # Swiss-army-knife of data styling and wrangling

library(fastDummies) # easy creation of dummy variables

library(dplyr) # See tidyverse

library(haven) # Import data from a variety of file types

library(broom) # easy tidying up of the output of various models and functions

library(colorspace) # Create and manipulate color schemes for charts etc

library(ggforce) # extra functions for ggplot2

library(extrafont)

# Import a few functions borrowed from ggpubr package.

source(here("scripts/ggpubr.R"))

```

#### Custom functions

This code block imports some functions from my scripts (found in the 'scripts/' directory). I moved these functions to external scripts to make the code in the present file cleaner and easier to read.

```{r custom_functions}

#| echo: false

#| include: false

# Import custom functions

source(here::here("scripts/custom_functions.R"))

replace_times_symbol <- function(x) {

# Check if any element in x matches the patterns

idx <- stringr::str_detect(x, "\\*")

# Replace only those elements

x[idx] <- stringr::str_replace_all(x[idx], "\\*", "&#xD7;")

# Convert each element to HTML

x <- sapply(x, gt::html)

return(x)

}

```

## Data operations

This code does some initial operations to the data.

```{r unweighted}

#| echo: false

#| include: false

#| cache: true

#| cache-vars: data.unweighted

# Initial manipulation of data

source(here::here("scripts/data_init_tidy.R"))

# This code block will create a variable for liquid assets to income ratio

data.unweighted <- data.unweighted %>%

mutate(hh_liquid_inc_ratio = hh_liquid/inc_disp_zeroed)

```

##### Quantiles

This code block will calculate quantiles using survey-weighted estimates for determining the cutoff values.

```{r quantiles}

#| echo: false

#| include: false

# Calculation of tob, alc and inc quantiles.

source(here::here("scripts/quantiles.R"), echo=FALSE)

```

```{r}

tables$income

```

```{r}

tables$tobacco

```

```{r}

tables$alcohol

```

##### Reliability

This code block calls an external script (within 'scripts/') which runs calculations including cronbach's alpha, assessing unidimensionality using IRT, then making a scree plot.

IMPORTANT!

This code block will take up to about 30 minutes to run due to the bootstrapping.

```{r reliability}

#| include: false

#| echo: false

#| cache: true

# Checks if the reliability data has been prepared earlier

if (file.exists(here::here("knit_files/reliability.rds"))) {

# If the reliability data has been prepared earlier, load it into object called 'reliability'

reliability <- readRDS(file = here::here("knit_files/reliability.rds"))

} else {

# If the reliability data has NOT been prepared earlier, run the calculations

# Might take up to 30 minutes to run.

source(here::here("scripts/reliability.R"))

}

```

This next code block gives the value (and 95% CI) for cronbachs alpha.

```{r format_inline_stats}

inline <- list("alpha" = list(

"est"=reliability$alpha$alpha,

"CI_low"=sprintf(reliability$alpha$ci[[1]], fmt = '%#.2f'),

"CI_high"=sprintf(reliability$alpha$ci[[2]], fmt = '%#.2f')))

inline

```

### Descriptive statistics

###### Descriptive stats setup

The following code blocks run some initial operations to prepare the descriptive tables.

```{r desc_tbl}

#| echo: false

#| include: false

fs_item_vars <- c("fin_stress_bills", "fin_stress_car", "fin_stress_pawn", "fin_stress_meals", "fin_stress_heat", "fin_stress_org", "fin_stress_family", "fin_stress_situ", "fin_stress_emgmoney")

data.unweighted <- data.unweighted %>% mutate(across(

.cols = all_of(fs_item_vars),

.fns = ~ as.integer(.)

)) %>%

mutate(across(

.cols = all_of(fs_item_vars),

.fns = ~ na_if(., 99)

)) %>%

mutate(

fin_stress_emgmoney = case_when(

fin_stress_emgmoney == 1 ~ 0,

fin_stress_emgmoney == 5 ~ 1

),

fin_stress_situ = case_when(

fin_stress_situ == 1 ~ 1,

fin_stress_situ == 2 ~ 0,

fin_stress_situ == 3 ~ 0

)

)

data.unweighted <- data.unweighted %>%

mutate(fin_stress_count_without_situ_emg = fin_stress_count - (fin_stress_emgmoney + fin_stress_situ))

## retaining the individual FS items (eg 'fin_') might have broken this code, will need to check. At first look it seems to run just fine.

data.unweighted <- dplyr::rename(data.unweighted, exp_alcohol_cat = exp_alc_cat, exp_tobacco_cat = exp_tob_cat) %>% mutate(inc_source=fct_collapse(inc_source, "Employment"=c("Employee"), "Government transfer"=c("Aged pensioner", "Other government transfer"), "Other"=c("Self-funded retiree", "Other"))

)

# Drop the rows with NA values for financial stress items and/or expenditure.

data.unweighted <- data.unweighted %>% drop_na(., c(starts_with("fin_stress"), starts_with("exp")))

source(here::here("scripts/descriptive.R"), echo=FALSE)

#individuals <- data.descriptive.subset %>%

# drop_na() %>% summarise(individuals=sum(as.integer(hh_num_persons))) %>% pull(individuals)

individuals <- data.descriptive.subset %>% summarise(individuals=sum(as.integer(hh_num_persons))) %>% pull(individuals)

```

```{r}

#| label: tbl-desc

#| cache: true

#| tbl-cap: "Unweighted sample characteristics"

#| include: false

# I believe this is no longer going to be included since this is the unweighted estimates. I believe we determined this is no longer relevant.

table_row_order <- c(

"fin_stress_count",

"exp_smoke_percent",

"exp_tobacco_cat",

"inc_disp_zeroed_equiv",

"inc_disp_cat",

"hh_asset_cat",

"inc_source",

"hh_disability",

"hh_tenure",

"hh_urban",

"hh_loneparent",

"exp_alcohol_binary",

"exp_gambling",

"ref_gender",

"ref_age_cat_collapse",

"ref_edu_collapse",

"ref_emp_occup_collapse"

)

tbl.desc.temp <- tables$descriptive$`_data`

tbl.desc.temp <- tbl.desc.temp %>%

mutate(groupname_col = case_when(

groupname_col=="Financial stress" ~ "Household",

groupname_col=="Tobacco expenditure proportion" ~ "Household",

groupname_col=="Equivalised household disposable income" ~ "Household",

groupname_col=="Household characteristics" ~ "Household",

groupname_col=="Reference person characteristics" ~ "Reference person",

),

tbl_id1 = case_when(groupname_col=="Household" ~ 1, groupname_col=="Reference person" ~ 2)) %>%

mutate(

order_index = match(variable, table_row_order)

) %>%

arrange(order_index, by_group = TRUE) %>%

mutate(label_variable_type = case_when(label == "Continuous" ~ "Continuous",

label == "Categorical" ~ "Categorical",

row_type == "label" ~ "Categorical",

TRUE ~ ""

)) %>%

relocate(c(var_label, label_variable_type,label, stat_0, stat_1, stat_2), .after = variable) %>%

mutate(var_label = case_when(

variable == "fin_stress_count" ~ "Financial stress count",

variable == "exp_smoke_percent" ~ "Tobacco expenditure",

variable == "exp_tobacco_cat" ~ "Tobacco expenditure",

variable == "inc_disp_zeroed_equiv" ~ "EHDI",

variable == "inc_disp_cat" ~ "EHDI",

variable == "hh_asset_cat" ~ "Wealth",

variable == "inc_source" ~ "Primary income source",

variable == "hh_disability" ~ "Chronic health condition",

variable == "hh_tenure" ~ "Tenure",

variable == "hh_urban" ~ "Captial city",

variable == "hh_loneparent" ~ "Lone parent",

variable == "exp_alcohol_binary" ~ "Alcohol expenditure",

variable == "exp_gambling" ~ "Gambling participation",

variable == "ref_gender" ~ "Gender",

variable == "ref_age_cat_collapse" ~ "Age",

variable == "ref_edu_collapse" ~ "Education",

variable == "ref_emp_occup_collapse" ~ "Occupation"),

label = case_when(

label == "No expenditure" ~ "None",

label == "Government transfer" ~ "Government",

label == "No post-secondary qualifications" ~ "No post-secondary",

TRUE ~ label

)) %>%

group_by(var_label) %>%

mutate(var_label = ifelse(row_number() > 1, "", var_label)) # This operation will make every value for var_label into an empty string except for the first appearance in the table.

# Replace 'label' with an empty string where both 'label' and 'label_variable_type' are "Continuous"

tbl.desc.temp.temp <- tbl.desc.temp %>%

mutate(label = ifelse(label == "Continuous" & label_variable_type == "Continuous", "", label))

# Remove rows where stat_0 is NA but not before copying the variable type to the next row.

rows_to_target <- which(is.na(tbl.desc.temp.temp$stat_0)) + 1

tbl.desc.temp.temp[rows_to_target,]$var_label <- tbl.desc.temp.temp[rows_to_target-1,]$var_label

tbl.desc.temp.temp[rows_to_target,]$label_variable_type <- tbl.desc.temp.temp[rows_to_target-1,]$label_variable_type

tbl.desc.temp.temp <- tbl.desc.temp.temp %>%

filter(!is.na(stat_0))

tbl.desc.temp.temp <- tbl.desc.temp.temp %>%

ungroup() %>%

group_by(groupname_col) %>%

gt() %>%

cols_hide(c(tbl_id1, variable, var_type, row_type, order_index)) %>%

tab_footnote(

"Median (IQR) for continuous; n (%) otherwise",

placement = "auto"

)

tbl.desc.temp.temp$`_spanners` <- tables$descriptive$`_spanners`

tbl.desc.temp.temp$`_options` <- tables$descriptive$`_options`

tbl.desc.temp.temp$`_substitutions` <- tables$descriptive$`_substitutions`

tbl.desc.temp.temp$`_substitutions`[[1]]$rows <- 1:44

tbl.desc.temp.temp%>% tab_style(style=cell_text(v_align="bottom",transform=NULL), locations=cells_row_groups()) %>%

tab_options(row_group.padding=3, row_group.padding.horizontal=2) %>%

cols_label(var_label = md("**Variable**"), label_variable_type = "", label = md("**Level**"), stat_0=md("**Overall**, N = 10,000"), stat_1=md("**No**, N = 8,327"),

stat_2=md("**Yes**, N = 1,673")) %>% tab_style(cell_text(style = 'italic'), cells_row_groups()) %>% tab_style_body(

style = cell_text(indent=px(10)),

columns = c("var_label"),

pattern = ".*"

) %>% tab_caption("Unweighted sample statistics")

#tables$descriptive

```

###### Unweighted

```{r v2_survey_desc}

#| label: tbl-desc-weighted

#| cache: true

#| tbl-cap: "Survey-weighted sample characteristics"

# Warn: this produces a table with N and % separated by a | symbol instead of actually appearing in separate columns.

desc.table.data <- data.subset %>% drop_na(., c(starts_with("fin_stress"), starts_with("exp"))) %>%

mutate(across(where(is.character),

~labelled::to_factor(.x,

levels = "values",

ordered = TRUE,

sort_levels = "auto",

labelled_only = FALSE,

strict = TRUE,

unclass = FALSE,

explicit_tagged_na = FALSE

)),

fin_stress_count = as.integer(fin_stress_count),

inc_disp_zeroed_equiv= inc_disp_zeroed_equiv/1000,

exp_tobacco_cat_5 = relevel(exp_tobacco_cat_5, ref="No expenditure"),

exp_smoke_proportion = exp_smoke_proportion*100,

exp_smoke_proportion_mort = exp_smoke_prop_combine *100,

exp_smoke_proportion_total = exp_smoke_prop_total *100,

exp_smoke_proportion_zero = as_factor(if_else(exp_smoke_proportion==0, 1, 0)),

exp_alcohol_proportion = exp_alcohol_proportion * 100,

exp_alcohol_cat = relevel(exp_alcohol_cat, ref="No expenditure"),

exp_alcohol_proportion_zero = as_factor(if_else(exp_alcohol_proportion==0, 1, 0)),

inc_disp_zeroed_equiv_100 = inc_disp_zeroed_equiv/100,

total_indicator = TRUE)

survey.v2.desc <- desc.table.data %>%

as_survey_rep(

type = "other",

combined_weights=TRUE,

repweights = starts_with("weight_reps_"),

weights = weight,

scale = (59 / 60),

rscale=rep(1,60),

mse=TRUE

)

tbl.descriptive.updated.unweighted <- tbl_summary(data=desc.table.data, type = list(exp_alcohol_binary ~ "categorical", exp_gambling ~ "categorical", hh_loneparent ~ "categorical"),

statistic = list(all_continuous() ~ "{median} ({p25}, {p75})", all_categorical() ~ "{n} ({p})", all_dichotomous() ~ "{n} ({p})"),

include=c(total_indicator, exp_tobacco_cat_5, inc_disp_cat, hh_liquid_cat, hh_tenure, hh_urban, ref_gender, ref_age_cat_collapse, ref_edu_collapse, hh_loneparent, exp_gambling, exp_alcohol_binary), label=c(total_indicator ~ "Total", exp_tobacco_cat_5 ~ "Tobacco expenditure",

hh_liquid_cat ~ "Liquidity",

ref_gender ~ "Gender",

exp_alcohol_binary = "Alcohol expenditure")) %>%

modify_header(stat_0 = "n (%)")

```

```{r descriptive_unweighted_table}

tbl.descriptive.updated.unweighted

```

###### Survey-weighted

```{r weighted_descriptive}

tbl.descriptive.updated.weighted <- tbl_svysummary(data=survey.v2.desc, type = list(exp_alcohol_binary ~ "categorical", exp_gambling ~ "categorical", hh_loneparent ~ "categorical"),

statistic = list(all_continuous() ~ "{median} ({p25}, {p75})", all_categorical() ~ "{p}", all_dichotomous() ~ "{p}"),

include=c(exp_tobacco_cat_5, inc_disp_cat, hh_liquid_cat, hh_tenure, hh_urban, ref_gender, ref_age_cat_collapse, ref_edu_collapse, hh_loneparent, exp_gambling, exp_alcohol_binary), label=c(exp_tobacco_cat_5 ~ "Tobacco expenditure",

hh_liquid_cat ~ "Liquidity",

ref_gender ~ "Gender",

exp_alcohol_binary = "Alcohol expenditure")) %>%

modify_header(stat_0 = "Weighted %")

tbl.descriptive.updated.weighted

```

```{r descriptive_weighted_table}

tbl.descriptive.updated.weighted

```

```{r inline_stats_printer}

inline_print <- function(tbl, stat_name, fmt = '%#.2f') {

out_est <- sprintf(tbl[tbl$stat == stat_name,]$est, fmt = fmt)

out_low <- sprintf(tbl[tbl$stat == stat_name,]$low, fmt = fmt)

out_upp <- sprintf(tbl[tbl$stat == stat_name,]$upp, fmt = fmt)

glue::glue("{out_est}% (95% CI: {out_low}, {out_upp})")

}

```

```{r calculate_svy_inline_stats}

# TES mean

survey.v2.desc.tobacco.mean <- survey.v2.desc %>% srvyr::group_by(exp_smoke_binary) %>% summarize(tes = survey_mean(exp_smoke_proportion, vartype="ci"))

inline.desc <- tibble(stat='tes.mean', est=survey.v2.desc.tobacco.mean[2,]$tes, low=survey.v2.desc.tobacco.mean[2,]$tes_low, upp=survey.v2.desc.tobacco.mean[2,]$tes_upp)

#TES median

survey.v2.desc.tobacco.median <- survey.v2.desc %>% srvyr::group_by(exp_smoke_binary) %>% summarize(tes = survey_median(exp_smoke_proportion, vartype="ci"))

inline.desc <- inline.desc %>% tibble::add_row(stat='tes.med', est=survey.v2.desc.tobacco.median[2,]$tes, low=survey.v2.desc.tobacco.median[2,]$tes_low, upp=survey.v2.desc.tobacco.median[2,]$tes_upp)

inline.tes.mean <- glue::glue("{survey.v2.desc.tobacco.mean[2,]$tes}% (95% CI: {survey.v2.desc.tobacco.mean[2,]$tes_low}, {survey.v2.desc.tobacco.mean[2,]$tes_upp})")

```

### Prevalence

```{r prev_table}

#| echo: false

source(here::here("scripts/prevalence.R"), echo=FALSE)

tbl.prev.temp <- tables$prevalence$`_data` %>% mutate(

label_order = case_when(

rowname_var == "Could not pay bills on time" ~ 1,

rowname_var == "Could not pay car registration or insurance on time" ~ 2,

rowname_var == "Pawned or sold something" ~ 3,

rowname_var == "Went without meals" ~ 4,

rowname_var == "Unable to heat home" ~ 5,

rowname_var == "Sought assistance from welfare/community organisation" ~ 6,

rowname_var == "Sought financial help from friends or family" ~ 7,

rowname_var == "Household typically spends more money that it gets" ~ 8,

rowname_var == "Not able to raise $2000 for something important within a week" ~ 9

)

) %>% arrange(label_order) %>% dplyr::select(-c(label_order))

prev_table_roboto <-tables$prevalence %>%opt_table_font(font="Roboto")

prev_table_roboto$`_data` <- tbl.prev.temp

prev_table_roboto %>% cleanse_table_html %>% tab_row_group(label="", rows=everything())

```

```{r prev_clean_est}

#| echo: false

prev <- tables[["prev_xtra"]] %>%

pivot_longer(cols = starts_with("prop"), names_to = c("stat", "finstress"),

names_pattern = "prop_?(.*)\\.([0-9]).*",

values_to = "prop"

) %>%

mutate(stat = dplyr::recode(na_if(stat,""), .missing="est")) %>%

pivot_wider(names_from = stat, values_from = prop) %>%

mutate(dplyr::across(c(est, low, upp), function(x) {gtsummary::style_percent(x,symbol = FALSE, digits=1)}))

```

```{r prev_items_plot}

#| cache: true

items.plot.data <- tables$prevalence$`_data` %>%

rename(prop.Overall_est = "prop.Overall",

prop.notob_est=`prop.No tobacco expenditure`,

prop.tob_est = `prop.Tobacco expenditure`,

prop.Overall_low = "prop_low.Overall",

prop.notob_low = `prop_low.No tobacco expenditure`,

prop.tob_low = `prop_low.Tobacco expenditure`,

prop.Overall_upp = "prop_upp.Overall",

prop.notob_upp = `prop_upp.No tobacco expenditure`,

prop.tob_upp = `prop_upp.Tobacco expenditure`) %>%

# Convert to long format, separating into type (prop, prop_low, prop_upp) and population (Overall, notob, tob)

pivot_longer(

cols = starts_with("prop"),

names_to = c("population", "type"),

names_pattern = "prop\\.([^\\_\\s]*)\\_*(.*)"

) %>%

pivot_wider(names_from=type, values_from=value) %>% mutate(rowname_mini = case_when(

rowname_var == "Could not pay bills on time" ~ "Unable to pay: bills",

rowname_var == "Could not pay car registration or insurance on time" ~ "Unable to pay: car",

rowname_var == "Household typically spends more money that it gets" ~ "Typically spends more than gets",

rowname_var == "Not able to raise $2000 for something important within a week" ~ "Unable to raise $2000",

rowname_var == "Pawned or sold something" ~ "Sold something",

rowname_var == "Sought assistance from welfare/community organisation" ~ "Sought help: organisation",

rowname_var == "Sought financial help from friends or family" ~ "Sought help: friends or family",

rowname_var == "Household typically spends more money that it gets" ~ "Spends more than gets",

rowname_var == "Unable to heat home" ~ "Unable to heat home",

rowname_var == "Went without meals" ~ "Went without meals",

))

# Convert the 'population' column to desired labels

items.plot.data$population <- recode(items.plot.data$population,

Overall = "Overall",

notob = "No",

tob = "Yes")

## This isnt really useful but I have included it because it

## makes it clear to the reader what the next step does

items.plot.data <- items.plot.data %>% mutate(

label_order = case_when(

rowname_mini == "Unable to pay: bills" ~ 1,

rowname_mini == "Unable to pay: car" ~ 2,

rowname_mini == "Sold something" ~ 3,

rowname_mini == "Went without meals" ~ 4,

rowname_mini == "Unable to heat home" ~ 5,

rowname_mini == "Sought help: organisation" ~ 6,

rowname_mini == "Sought help: friends or family" ~ 7,

rowname_mini == "Spends more than gets" ~ 8,

rowname_mini == "Unable to raise $2000" ~ 9

)

)

## This is the next step, which makes a list of the item labels in the correct order

ordered_strings <- items.plot.data %>%

arrange(label_order) %>%

pull(rowname_mini)

item.plot <- ggplot(subset(items.plot.data, population!="Overall"), aes(x=est, y=rowname_mini, group=population, color=population, xmin=low, xmax=upp)) + scale_x_continuous(labels = scales::percent) + colorspace::scale_color_discrete_sequential(palette = "GreenMono", rev = TRUE, limits=c("dummylvl", "No", "Yes"), breaks=c("No", "Yes")) + geom_hline(aes(yintercept =rowname_mini),

linetype = "dashed",

color = "lightgray") + geom_errorbar(colour='black',

width=.2, size=0.7) + geom_point(size=4) +

theme_pubr() +

labs_pubr() +

labs(x = "Prevalence (%)",

y = "",

color = "Tobacco status") +

theme_Publication(base_size = 14) +

scale_y_discrete(expand=c(0.15,0.15), limits=rev(unique(ordered_strings))) + theme(axis.line.y = element_blank(),

axis.ticks.y = element_blank())

```

```{r}

item.plot

```

```{r prev_plot}

#| echo: false

#| cache: true

#| fig-cap: "Financial stresses in households with and without tobacco expenditure"

knitr::opts_chunk$set(fig.showtext=TRUE)

dist.plot <- tables$prevalence_old[["_data"]] %>%

#dplyr::select(-c("unweighted", "svytotal")) %>%

#filter(is.na(exp_smoke_binary)) %>%

pivot_longer(cols = starts_with("prop"), names_to = c("stat", "finstress"),

names_pattern = "prop_?(.*)\\.(.)",

values_to = "prop"

) %>%

filter(exp_smoke_binary != "Overall" & finstress %in% c("0", "1", "2", "3", "4", "5")) %>%

mutate(stat = dplyr::recode(na_if(stat,""), .missing="est")) %>%

pivot_wider(names_from = stat, values_from = prop) %>%

mutate(exp_smoke_binary=fct_expand(as_factor(exp_smoke_binary), "dummylvl")) %>%

ggplot(aes(x=finstress, y=est, fill = exp_smoke_binary)) +

geom_bar(stat="identity",

colour="black", # Black outline for all

position=position_dodge(), size = 0)+# Put bars side-by-side instead of stacked

geom_errorbar(aes(ymin=low, ymax=upp),

position=position_dodge(.9),

width=.75, size=0.3) +

scale_x_discrete(labels=c("0" = "0",

"1" = "1",

"2" = "2",

"3" = "3",

"4" = "4",

"5" = "5+")) +

scale_y_continuous(labels = scales::percent) +

colorspace::scale_fill_discrete_sequential(palette = "GreenMono", rev = TRUE, limits=c("dummylvl", "No", "Yes"), breaks=c("No", "Yes")) +

theme_pubr() +

labs_pubr() +

labs(x = "Financial stress score",

y = "Prevalence (%)",

fill = "Tobacco status") +

theme_Publication(base_size = 14)

```

```{r}

dist.plot

```

```{r prev_cowplot}

#| cache: true

#| label: fig-prev

#| fig-cap: "Estimated prevalence of financial stress indicators by household tobacco status: (A) distribution of total scores, (B) by item"

library("cowplot")

library(grid)

prev.legend <- get_legend(item.plot + theme(legend.position="bottom", legend.direction="vertical", legend.key = element_rect(fill = "transparent")))

cowplot.prev.plot <- plot_grid(

dist.plot+theme(axis.title.y = element_text(margin = margin(r = 10)), axis.title.x = element_text(margin = margin(t = 10)),

legend.position="none"),

item.plot +labs(caption = "Plot A: Due to low counts for scores 6-9, scores of ≥5 are displayed as a single group to protect data privacy")+theme(plot.caption=element_text(size=8, margin=margin(t = 20, r = 0, b = 0, l = 0, unit = "pt")), legend.position="none", axis.text.y = element_text(face='bold', size = 11), axis.title.x = element_text(margin = margin(t = 10))),

labels="AUTO", axis = "tb", nrow = 2, ncol=1, rel_heights = c(1.5, 1))

#cowplot.prev.plot <- add_sub(cowplot.prev.plot, "Plot A displays scores of ≥5 as a single group to protect data privacy due to low counts for scores 6-9", size=10, x = 0, hjust = 0)

cowplot.prev.plot <- ggdraw(cowplot.prev.plot) + draw_plot(prev.legend, .4, .4, 0.5, 0.5)

aspect_ratio_prev <- 0.877

if (interactive()) {

cowplot::ggsave2(here::here("knit_files/prev.png"), device=ragg::agg_png, cowplot.prev.plot, height = 4, width=4*aspect_ratio_prev, dpi=600, scale=2, background = "white")

} else {

knitr::include_graphics(here::here('knit_files/prev.png'))

}

```

```{r}

cowplot.prev.plot

```

```{r format_inline_stats_prev}

inline$prev_p <- list("chi" = sprintf(stat.prev$statistic, fmt = '%#.1f'), "df" =as.integer(stat.prev$parameter), "p" = gtsummary::style_pvalue(stat.prev$p.value, prepend_p=TRUE))

num_to_million <- function(num) {

# Divide the number by a million and round to 2 decimal places

num_in_million <- round(num / 1e6, 2)

# Convert the number to a string and return it

return(as.character(num_in_million))

}

tob_total <- tbl_prev_tobonly %>% mutate(mil.tot = num_to_million(svytotal), mil.low = num_to_million(svytotal_low), mil.upp = num_to_million(svytotal_upp))

```

## Regression

```{r regression_setup}

#| cache: true

survey.simple <- data.subset %>% drop_na(., c(starts_with("fin_stress"), starts_with("exp"))) %>%

mutate(across(where(is.character),

~labelled::to_factor(.x,

levels = "values",

ordered = TRUE,

sort_levels = "auto",

labelled_only = FALSE,

strict = TRUE,

unclass = FALSE,

explicit_tagged_na = FALSE

)),

fin_stress_count = as.integer(fin_stress_count),

inc_disp_zeroed_equiv= inc_disp_zeroed_equiv/1000,

exp_tobacco_cat_5 = relevel(exp_tobacco_cat_5, ref="No expenditure"),

exp_smoke_proportion = exp_smoke_proportion*100,

exp_smoke_proportion_mort = exp_smoke_prop_combine *100,

exp_smoke_proportion_total = exp_smoke_prop_total *100,

exp_smoke_proportion_zero = as_factor(if_else(exp_smoke_proportion==0, 1, 0)),

exp_alcohol_proportion = exp_alcohol_proportion * 100,

exp_alcohol_cat = relevel(exp_alcohol_cat, ref="No expenditure"),

exp_alcohol_proportion_zero = as_factor(if_else(exp_alcohol_proportion==0, 1, 0)),

inc_disp_zeroed_equiv_100 = inc_disp_zeroed_equiv/100) %>%

#mutate(across(c(exp_tobacco_cat,exp_alcohol_cat,inc_disp_cat,hh_asset_cat,ref_age_cat_collapse,ref_edu_collapse,ref_emp_occup_collapse), as.ordered)) %>%

as_survey_design(

weights = weight)

weights <- stats::weights(survey.simple)

weights <- weights/mean(weights)

```

##### Poisson

The purpose of running these Poission models is to check whether the model is overdispersed (relative to the Poisson distribution). We do find evidence of overdispersion, which is why we then fit a negative binomial model instead.

###### Model 1

```{r poisson_for_overdispersion_simplified_model1}

simplified.regression.poi <- glmmTMB::glmmTMB(fin_stress_count~exp_tobacco_cat_5+inc_disp_cat+ref_gender+ref_age_cat_collapse,family = poisson(),data=survey.simple$variables, weights=weights)

performance::check_overdispersion(simplified.regression.poi)

```

###### Model 2

```{r poisson_for_overdispersion_simplified_model2}

simplified.regression.poi <- glmmTMB::glmmTMB(fin_stress_count~exp_tobacco_cat_5+inc_disp_cat+ref_gender+ref_age_cat_collapse+hh_liquid_cat+hh_tenure+hh_urban+ref_edu_collapse+hh_loneparent,family = poisson(),data=survey.simple$variables, weights=weights)

performance::check_overdispersion(simplified.regression.poi)

```

###### Model 3

```{r poisson_for_overdispersion_simplified_model3}

simplified.regression.poi <- glmmTMB::glmmTMB(fin_stress_count~exp_tobacco_cat_5+inc_disp_cat+hh_liquid_cat+hh_tenure+hh_urban+ref_gender+ref_age_cat_collapse+ref_edu_collapse+hh_loneparent+exp_gambling+exp_alcohol_proportion_zero,family = poisson(),data=survey.simple$variables, weights=weights)

performance::check_overdispersion(simplified.regression.poi)

```

##### Main regressions

The main models use a GLM with negative binomial distribution.

```{r main_regression}

simplified.regression.nb.cont.1 <- glmmTMB::glmmTMB(fin_stress_count~exp_tobacco_cat_5+inc_disp_cat+ref_gender+ref_age_cat_collapse,family = glmmTMB::nbinom2(),data=survey.simple$variables, weights=weights)

simplified.regression.nb.cont.2 <- glmmTMB::glmmTMB(fin_stress_count~exp_tobacco_cat_5+inc_disp_cat+hh_liquid_cat+hh_tenure+hh_urban+ref_gender+ref_age_cat_collapse+ref_edu_collapse+hh_loneparent,family = glmmTMB::nbinom2(),data=survey.simple$variables, weights=weights)

simplified.regression.nb.cont.3 <- glmmTMB::glmmTMB(fin_stress_count~exp_tobacco_cat_5+inc_disp_cat+hh_liquid_cat+hh_tenure+hh_urban+ref_gender+ref_age_cat_collapse+ref_edu_collapse+hh_loneparent+exp_gambling+exp_alcohol_proportion_zero,family = glmmTMB::nbinom2(),data=survey.simple$variables, weights=weights)

tbl.model1 <- gtsummary::tbl_regression(simplified.regression.nb.cont.1, exponentiate=TRUE) %>% add_glance_table(

include = c(AIC, nobs)

)

tbl.model2 <- gtsummary::tbl_regression(simplified.regression.nb.cont.2, exponentiate=TRUE)%>% add_glance_table(

include = c(AIC, nobs)

)

tbl.model3 <- gtsummary::tbl_regression(simplified.regression.nb.cont.3, exponentiate=TRUE)%>% add_glance_table(

include = c(AIC, nobs)

)

tbls.all <- tbl_merge(list(tbl.model1, tbl.model2, tbl.model3))

tbls.all

```

##### Main regressions

The main models use a GLM with negative binomial distribution with a bivariable column (table 4).

```{r updated_regression_binary}

# Multivariable models

simplified.regression.nb.cont.1 <- glmmTMB::glmmTMB(fin_stress_count ~ exp_tobacco_cat_5 + inc_disp_cat + ref_gender + ref_age_cat_collapse,

family = glmmTMB::nbinom2(),

data = survey.simple$variables,

weights = weights)

simplified.regression.nb.cont.2 <- glmmTMB::glmmTMB(fin_stress_count ~ exp_tobacco_cat_5 + inc_disp_cat + hh_liquid_cat + hh_tenure + hh_urban + ref_gender + ref_age_cat_collapse + ref_edu_collapse + hh_loneparent,

family = glmmTMB::nbinom2(),

data = survey.simple$variables,

weights = weights)

simplified.regression.nb.cont.3 <- glmmTMB::glmmTMB(fin_stress_count ~ exp_tobacco_cat_5 + inc_disp_cat + hh_liquid_cat + hh_tenure + hh_urban + ref_gender + ref_age_cat_collapse + ref_edu_collapse + hh_loneparent + exp_gambling + exp_alcohol_proportion_zero,

family = glmmTMB::nbinom2(),

data = survey.simple$variables,

weights = weights)

# Bivariable models for all predictors

simplified.regression.nb.cont.bivar1 <- glmmTMB::glmmTMB(fin_stress_count ~ exp_tobacco_cat_5,

family = glmmTMB::nbinom2(),

data = survey.simple$variables,

weights = weights)

simplified.regression.nb.cont.bivar2 <- glmmTMB::glmmTMB(fin_stress_count ~ inc_disp_cat,

family = glmmTMB::nbinom2(),

data = survey.simple$variables,

weights = weights)

simplified.regression.nb.cont.bivar3 <- glmmTMB::glmmTMB(fin_stress_count ~ hh_liquid_cat,

family = glmmTMB::nbinom2(),

data = survey.simple$variables,

weights = weights)

simplified.regression.nb.cont.bivar4 <- glmmTMB::glmmTMB(fin_stress_count ~ hh_tenure,

family = glmmTMB::nbinom2(),

data = survey.simple$variables,

weights = weights)

simplified.regression.nb.cont.bivar5 <- glmmTMB::glmmTMB(fin_stress_count ~ hh_urban,

family = glmmTMB::nbinom2(),

data = survey.simple$variables,

weights = weights)

simplified.regression.nb.cont.bivar6 <- glmmTMB::glmmTMB(fin_stress_count ~ ref_gender,

family = glmmTMB::nbinom2(),

data = survey.simple$variables,

weights = weights)

simplified.regression.nb.cont.bivar7 <- glmmTMB::glmmTMB(fin_stress_count ~ ref_age_cat_collapse,

family = glmmTMB::nbinom2(),

data = survey.simple$variables,

weights = weights)

simplified.regression.nb.cont.bivar8 <- glmmTMB::glmmTMB(fin_stress_count ~ ref_edu_collapse,

family = glmmTMB::nbinom2(),

data = survey.simple$variables,

weights = weights)

simplified.regression.nb.cont.bivar9 <- glmmTMB::glmmTMB(fin_stress_count ~ hh_loneparent,

family = glmmTMB::nbinom2(),

data = survey.simple$variables,

weights = weights)

simplified.regression.nb.cont.bivar10 <- glmmTMB::glmmTMB(fin_stress_count ~ exp_gambling,

family = glmmTMB::nbinom2(),

data = survey.simple$variables,

weights = weights)

simplified.regression.nb.cont.bivar11 <- glmmTMB::glmmTMB(fin_stress_count ~ exp_alcohol_proportion_zero,

family = glmmTMB::nbinom2(),

data = survey.simple$variables,

weights = weights)

# Creating regression tables for each multivariable model

tbl.model1 <- gtsummary::tbl_regression(simplified.regression.nb.cont.1, exponentiate = TRUE) %>%

add_glance_table(include = c(AIC, nobs))

tbl.model2 <- gtsummary::tbl_regression(simplified.regression.nb.cont.2, exponentiate = TRUE) %>%

add_glance_table(include = c(AIC, nobs))

tbl.model3 <- gtsummary::tbl_regression(simplified.regression.nb.cont.3, exponentiate = TRUE) %>%

add_glance_table(include = c(AIC, nobs))

tbl.bivar1 <- gtsummary::tbl_regression(simplified.regression.nb.cont.bivar1, exponentiate = TRUE)

tbl.bivar2 <- gtsummary::tbl_regression(simplified.regression.nb.cont.bivar2, exponentiate = TRUE)

tbl.bivar3 <- gtsummary::tbl_regression(simplified.regression.nb.cont.bivar3, exponentiate = TRUE)

tbl.bivar4 <- gtsummary::tbl_regression(simplified.regression.nb.cont.bivar4, exponentiate = TRUE)

tbl.bivar5 <- gtsummary::tbl_regression(simplified.regression.nb.cont.bivar5, exponentiate = TRUE)

tbl.bivar6 <- gtsummary::tbl_regression(simplified.regression.nb.cont.bivar6, exponentiate = TRUE)

tbl.bivar7 <- gtsummary::tbl_regression(simplified.regression.nb.cont.bivar7, exponentiate = TRUE)

tbl.bivar8 <- gtsummary::tbl_regression(simplified.regression.nb.cont.bivar8, exponentiate = TRUE)

tbl.bivar9 <- gtsummary::tbl_regression(simplified.regression.nb.cont.bivar9, exponentiate = TRUE)

tbl.bivar10 <- gtsummary::tbl_regression(simplified.regression.nb.cont.bivar10, exponentiate = TRUE)

tbl.bivar11 <- gtsummary::tbl_regression(simplified.regression.nb.cont.bivar11, exponentiate = TRUE)

# Combine all bivariable tables

tbls.bivar <- tbl_stack(list(tbl.bivar1, tbl.bivar2, tbl.bivar3, tbl.bivar4, tbl.bivar5, tbl.bivar6, tbl.bivar7, tbl.bivar8, tbl.bivar9, tbl.bivar10, tbl.bivar11))

# Combine all multivariable and bivariable tables

tbls.all <- tbl_merge(list(tbl.model1, tbl.model2, tbl.model3, tbls.bivar))

# Display the combined table

tbls.all

# Load necessary libraries

library(gtsummary)

library(flextable)

library(officer)

# Assuming tbls.all is your final gtsummary table

flex_table <- as_flex_table(tbls.all)

save_as_docx(flex_table, path = "table_output.docx")

```

##### Supplementary regression models

#For inclusion in the supplementary materials.

###### 1+ vs none (of 9 indicators)

These are logistic regressions. Outcome of these models is "one or more indicators" vs "zero indicators". Total of 9 possible indicators.

```{r updated_regression_binary}

simplified.regression.binary.1 <- glmmTMB::glmmTMB(fin_stress_binary~exp_tobacco_cat_5+inc_disp_cat+ref_gender+ref_age_cat_collapse,family = "binomial",data=survey.simple$variables, weights=weights)

simplified.regression.binary.2 <- glmmTMB::glmmTMB(fin_stress_binary~exp_tobacco_cat_5+inc_disp_cat+hh_liquid_cat+hh_tenure+hh_urban+ref_gender+ref_age_cat_collapse+ref_edu_collapse+hh_loneparent,family = "binomial",data=survey.simple$variables, weights=weights)

simplified.regression.binary.3 <- glmmTMB::glmmTMB(fin_stress_binary~exp_tobacco_cat_5+inc_disp_cat+hh_liquid_cat+hh_tenure+hh_urban+ref_gender+ref_age_cat_collapse+ref_edu_collapse+hh_loneparent+exp_gambling+exp_alcohol_proportion_zero,family = "binomial",data=survey.simple$variables, weights=weights)

tbls.binary.all <- tbl_merge(list(gtsummary::tbl_regression(simplified.regression.binary.1, exponentiate=TRUE) %>% add_glance_table(

include = c(AIC, nobs)

), gtsummary::tbl_regression(simplified.regression.binary.2, exponentiate=TRUE) %>% add_glance_table(

include = c(AIC, nobs)

), gtsummary::tbl_regression(simplified.regression.binary.3, exponentiate=TRUE) %>% add_glance_table(

include = c(AIC, nobs)

)))

tbls.binary.all

```

###### 1+ vs none (of 7 indicators)

These are logistic regressions. Outcome of these models is "one or more indicators" vs "zero indicators". Total of 7 possible indicators.

```{r updated_regression_7_item}

simplified.regression.nb.cont.7item.1 <- glmmTMB::glmmTMB(fin_stress_count_without_situ_emg~exp_tobacco_cat_5+inc_disp_cat+ref_gender+ref_age_cat_collapse,family = glmmTMB::nbinom2(),data=survey.simple$variables, weights=weights)

simplified.regression.nb.cont.7item.2 <- glmmTMB::glmmTMB(fin_stress_count_without_situ_emg~exp_tobacco_cat_5+inc_disp_cat+hh_liquid_cat+hh_tenure+hh_urban+ref_gender+ref_age_cat_collapse+ref_edu_collapse+hh_loneparent,family = glmmTMB::nbinom2(),data=survey.simple$variables, weights=weights)

simplified.regression.nb.cont.7item.3 <- glmmTMB::glmmTMB(fin_stress_count_without_situ_emg~exp_tobacco_cat_5+inc_disp_cat+hh_liquid_cat+hh_tenure+hh_urban+ref_gender+ref_age_cat_collapse+ref_edu_collapse+hh_loneparent+exp_gambling+exp_alcohol_proportion_zero,family = glmmTMB::nbinom2(),data=survey.simple$variables, weights=weights)

tbls.7item.all <- tbl_merge(list(gtsummary::tbl_regression(simplified.regression.nb.cont.7item.1, exponentiate=TRUE) %>% add_glance_table(

include = c(AIC, nobs)

), gtsummary::tbl_regression(simplified.regression.nb.cont.7item.2, exponentiate=TRUE) %>% add_glance_table(

include = c(AIC, nobs)

), gtsummary::tbl_regression(simplified.regression.nb.cont.7item.3, exponentiate=TRUE) %>% add_glance_table(

include = c(AIC, nobs)

)))

tbls.7item.all

```

###### Went without meals

These models are logistic regression predicting the 'went without meals' item ONLY.

```{r updated_regression_meals_only}

simplified.regression.binary.meals.1 <- glmmTMB::glmmTMB(fin_stress_meals~exp_tobacco_cat_5+inc_disp_cat+ref_gender+ref_age_cat_collapse,family = "binomial",data=survey.simple$variables, weights=weights)

simplified.regression.binary.meals.2 <- glmmTMB::glmmTMB(fin_stress_meals~exp_tobacco_cat_5+inc_disp_cat+hh_liquid_cat+hh_tenure+hh_urban+ref_gender+ref_age_cat_collapse+ref_edu_collapse+hh_loneparent,family = "binomial",data=survey.simple$variables, weights=weights)

simplified.regression.binary.meals.3 <- glmmTMB::glmmTMB(fin_stress_meals~exp_tobacco_cat_5+inc_disp_cat+hh_liquid_cat+hh_tenure+hh_urban+ref_gender+ref_age_cat_collapse+ref_edu_collapse+hh_loneparent+exp_gambling+exp_alcohol_proportion_zero,family = "binomial",data=survey.simple$variables, weights=weights)

tbls.binary.meals.all <- tbl_merge(list(gtsummary::tbl_regression(simplified.regression.binary.meals.1, exponentiate=TRUE) %>% add_glance_table(

include = c(AIC, nobs)

), gtsummary::tbl_regression(simplified.regression.binary.meals.2, exponentiate=TRUE) %>% add_glance_table(

include = c(AIC, nobs)

), gtsummary::tbl_regression(simplified.regression.binary.meals.3, exponentiate=TRUE) %>% add_glance_table(

include = c(AIC, nobs)

)))

tbls.binary.meals.all

```

###### Negative binomial (excl. imputed)

These models are identical to the main (negative binomial) models, but instead of including all households, we only include the households without data imputation (complete cases).

```{r regression_exclude_imputed}

# 6403 observations

survey.simple.noimput <- data.subset %>% drop_na(., c(starts_with("fin_stress"), starts_with("exp"))) %>% filter(!has_imput) %>% mutate(across(where(is.character),

~labelled::to_factor(.x,

levels = "values",

ordered = TRUE,

sort_levels = "auto",

labelled_only = FALSE,

strict = TRUE,

unclass = FALSE,

explicit_tagged_na = FALSE

)),

fin_stress_count = as.integer(fin_stress_count),

inc_disp_zeroed_equiv = inc_disp_zeroed_equiv/1000,

exp_smoke_proportion = exp_smoke_proportion * 100,

exp_smoke_proportion_mort = exp_smoke_prop_combine *100,

exp_smoke_proportion_total = exp_smoke_prop_total *100,

exp_smoke_proportion_zero = as_factor(if_else(exp_smoke_proportion==0, 1, 0)),

exp_alcohol_proportion = exp_alcohol_proportion * 100,

exp_alcohol_proportion_zero = as_factor(if_else(exp_alcohol_proportion==0, 1, 0)),

inc_disp_zeroed_equiv_100 = inc_disp_zeroed_equiv/100) %>%

#mutate(across(c(exp_tobacco_cat,exp_alcohol_cat,inc_disp_cat,hh_asset_cat,ref_age_cat_collapse,ref_edu_collapse,ref_emp_occup_collapse), as.ordered)) %>%

as_survey_design(

weights = weight)

weights.noimput <- stats::weights(survey.simple.noimput)

weights.noimput <- weights.noimput/mean(weights.noimput)

simplified.regression.nb.cont.1.noimput <- glmmTMB::glmmTMB(fin_stress_count~exp_tobacco_cat_5+inc_disp_cat+ref_gender+ref_age_cat_collapse,family = glmmTMB::nbinom2(),data=

survey.simple.noimput$variables, weights=weights.noimput)

simplified.regression.nb.cont.2.noimput <- glmmTMB::glmmTMB(fin_stress_count~exp_tobacco_cat_5+inc_disp_cat+hh_liquid_cat+hh_tenure+hh_urban+ref_gender+ref_age_cat_collapse+ref_edu_collapse+hh_loneparent,family = glmmTMB::nbinom2(),data=survey.simple.noimput$variables, weights=weights.noimput)

simplified.regression.nb.cont.3.noimput <- glmmTMB::glmmTMB(fin_stress_count~exp_tobacco_cat_5+inc_disp_cat+hh_liquid_cat+hh_tenure+hh_urban+ref_gender+ref_age_cat_collapse+ref_edu_collapse+hh_loneparent+exp_gambling+exp_alcohol_proportion_zero,family = glmmTMB::nbinom2(),data=survey.simple.noimput$variables, weights=weights.noimput)

tbl.model1.noimput <- gtsummary::tbl_regression(simplified.regression.nb.cont.1.noimput, exponentiate=TRUE) %>% add_glance_table(

include = c(AIC, nobs)

)

tbl.model2.noimput <- gtsummary::tbl_regression(simplified.regression.nb.cont.2.noimput, exponentiate=TRUE)%>% add_glance_table(

include = c(AIC, nobs)

)

tbl.model3.noimput <- gtsummary::tbl_regression(simplified.regression.nb.cont.3.noimput, exponentiate=TRUE)%>% add_glance_table(

include = c(AIC, nobs)

)

tbls.all.noimput <- tbl_merge(list(tbl.model1.noimput, tbl.model2.noimput, tbl.model3.noimput))

tbls.all.noimput

```

## Model diagnostics

The following code blocks check assumptions of the regression models and check model fit.

#### Check for (multi)colinearity

The following code block tests the predictors of the full main model (the negative binomial model with all predictors) for multicolinearity.

```{r check_collinearity}

#| include: false

performance::check_collinearity(

simplified.regression.nb.cont.3)

```

#### Check for zero-inflation

The following code block checks the full main model (the negative binomial model with all predictors) for evidence of zero-inflation.

```{r check_zeroinflation_simplified_model3}

performance::check_zeroinflation(simplified.regression.nb.cont.3)

```

#### Assessment of model fit

The following code block uses the likelihood ratio test to compare the fit of the main negative binomial models (each with the same outcome variable but with progressively greater number of predictors).

```{r check_lrt}

#| include: false

performance::test_lrt(

simplified.regression.nb.cont.1,

simplified.regression.nb.cont.2,

simplified.regression.nb.cont.3)

```
