# Supplementary material for: Financial Stress and Tobacco Expenditure in Australian Households: A Cross-Sectional Analysis of Prevalence and Association Across Wealth and Income Levels
Source: Nicotine Tob Res. 2025 May 13;28(1):117–27. doi: 10.1093/ntr/ntaf102 (PMC12723215; doi:10.1093/ntr/ntaf102)
Supplement: ntaf102_suppl_Supplementary_Materials [file ntaf102_suppl_supplementary_materials.docx]

## Sensitivity analyses

Table S1: Logistic regression models predicting financial stress (≥1 items vs 0)

|  | **Multivariable model (1)** | | |  | **Multivariable model (2)** | | |  | **Multivariable model (3)** | | |
| --- | --- | --- | --- | --- | --- | --- | --- | --- | --- | --- | --- |
|  | **OR***^1^* | **95% CI***^2^* | **p-value** |  | **OR***^1^* | **95% CI***^2^* | **p-value** |  | **OR***^1^* | **95% CI***^2^* | **p-value** |
| Tobacco expenditure ^a^ |  |  |  |  |  |  |  |  |  |  |  |
| None | Ref. |  |  |  | Ref. |  |  |  | Ref. |  |  |
| 1 (Lowest) | 1.88 | 1.48, 2.39 | <0.001 |  | 1.53 | 1.19, 1.97 | <0.001 |  | 1.54 | 1.20, 1.99 | <0.001 |
| 2 | 2.36 | 1.87, 2.97 | <0.001 |  | 1.71 | 1.33, 2.20 | <0.001 |  | 1.72 | 1.34, 2.21 | <0.001 |
| 3 | 1.87 | 1.49, 2.36 | <0.001 |  | 1.35 | 1.05, 1.73 | 0.017 |  | 1.36 | 1.06, 1.74 | 0.015 |
| 4 | 2.28 | 1.81, 2.86 | <0.001 |  | 1.62 | 1.26, 2.07 | <0.001 |  | 1.63 | 1.27, 2.09 | <0.001 |
| 5 (Highest) | 2.83 | 2.25, 3.55 | <0.001 |  | 1.90 | 1.48, 2.45 | <0.001 |  | 1.92 | 1.49, 2.47 | <0.001 |
| Equiv. disposable income ^b^ |  |  |  |  |  |  |  |  |  |  |  |
| 5 (Highest) | Ref. |  |  |  | Ref. |  |  |  | Ref. |  |  |
| 4 | 2.30 | 1.92, 2.75 | <0.001 |  | 1.90 | 1.58, 2.30 | <0.001 |  | 1.90 | 1.57, 2.30 | <0.001 |
| 3 | 3.55 | 2.98, 4.23 | <0.001 |  | 2.96 | 2.46, 3.56 | <0.001 |  | 2.94 | 2.44, 3.54 | <0.001 |
| 2 | 5.72 | 4.80, 6.81 | <0.001 |  | 4.29 | 3.56, 5.17 | <0.001 |  | 4.24 | 3.51, 5.12 | <0.001 |
| 1 (Lowest) | 8.63 | 7.23, 10.3 | <0.001 |  | 7.13 | 5.89, 8.63 | <0.001 |  | 7.01 | 5.77, 8.51 | <0.001 |
| Gender |  |  |  |  |  |  |  |  |  |  |  |
| Male | Ref. |  |  |  | Ref. |  |  |  | Ref. |  |  |
| Female | 1.24 | 1.13, 1.36 | <0.001 |  | 1.17 | 1.05, 1.30 | 0.003 |  | 1.17 | 1.05, 1.30 | 0.003 |
| Age |  |  |  |  |  |  |  |  |  |  |  |
| 15-34 | Ref. |  |  |  | Ref. |  |  |  | Ref. |  |  |
| 35-54 | 0.82 | 0.73, 0.92 | 0.001 |  | 1.03 | 0.90, 1.18 | 0.7 |  | 1.03 | 0.90, 1.18 | 0.6 |
| 55+ | 0.39 | 0.34, 0.44 | <0.001 |  | 0.93 | 0.79, 1.08 | 0.3 |  | 0.93 | 0.79, 1.09 | 0.4 |
| Liquidity |  |  |  |  |  |  |  |  |  |  |  |
| Low |  |  |  |  | Ref. |  |  |  | Ref. |  |  |
| Middle |  |  |  |  | 0.33 | 0.29, 0.37 | <0.001 |  | 0.33 | 0.29, 0.37 | <0.001 |
| High |  |  |  |  | 0.23 | 0.20, 0.26 | <0.001 |  | 0.23 | 0.20, 0.26 | <0.001 |
| Tenure |  |  |  |  |  |  |  |  |  |  |  |
| Owned outright |  |  |  |  | Ref. |  |  |  | Ref. |  |  |
| Mortgaged |  |  |  |  | 1.76 | 1.51, 2.06 | <0.001 |  | 1.76 | 1.51, 2.06 | <0.001 |
| Rented |  |  |  |  | 2.89 | 2.47, 3.38 | <0.001 |  | 2.88 | 2.46, 3.38 | <0.001 |
| Other |  |  |  |  | 1.98 | 1.43, 2.74 | <0.001 |  | 1.97 | 1.43, 2.73 | <0.001 |
| Household location |  |  |  |  |  |  |  |  |  |  |  |
| Greater capital city |  |  |  |  | Ref. |  |  |  | Ref. |  |  |
| Rest of state |  |  |  |  | 0.90 | 0.81, 1.00 | 0.053 |  | 0.90 | 0.82, 1.00 | 0.059 |
| Education |  |  |  |  |  |  |  |  |  |  |  |
| Degree or higher |  |  |  |  | Ref. |  |  |  | Ref. |  |  |
| Certificate to Diploma |  |  |  |  | 1.16 | 1.01, 1.32 | 0.036 |  | 1.16 | 1.01, 1.32 | 0.033 |
| No post-secondary qualifications |  |  |  |  | 1.14 | 0.99, 1.31 | 0.067 |  | 1.14 | 0.99, 1.31 | 0.066 |
| Lone parent |  |  |  |  |  |  |  |  |  |  |  |
| No |  |  |  |  | Ref. |  |  |  | Ref. |  |  |
| Yes |  |  |  |  | 1.89 | 1.59, 2.26 | <0.001 |  | 1.89 | 1.58, 2.25 | <0.001 |
| Gambling |  |  |  |  |  |  |  |  |  |  |  |
| No |  |  |  |  |  |  |  |  | Ref. |  |  |
| Yes |  |  |  |  |  |  |  |  | 0.98 | 0.87, 1.11 | 0.8 |
| Alcohol |  |  |  |  |  |  |  |  |  |  |  |
| Yes |  |  |  |  |  |  |  |  | Ref. |  |  |
| No |  |  |  |  |  |  |  |  | 1.04 | 0.94, 1.16 | 0.4 |
| *n* obs. | 10,036 |  |  |  | 10,036 |  |  |  | 10,036 |  |  |
| *Note:* ^1^CI = Confidence Interval; RR = Risk Ratio; ^a^ Tobacco expenditure levels = either zero expenditure or into five equally distributed household expenditure quintiles; ^b^ Disposable income = gross income minus income tax and levies, i.e. the net income available for consumption and saving ^#^ Gender, age and education are the characteristics of the household referenced person, who is a person chosen to represent a household in a survey | | | | | | | | | | | |

The following regression model uses logistic regression but excludes two financial stress items from the definition of financial stress: “Household typically spends more money than it gets” and “not able to raise $2000 for something important within a week”. Other published evidence using the HES sometimes excludes these items, using only the remaining seven items to define financial stress.

Table S2: Logistic regression models predicting financial stress, seven item instrument (≥1 items vs 0)

|  | **Multivariable model (1)** | | |  | **Multivariable model (2)** | | |  | **Multivariable model (3)** | | |
| --- | --- | --- | --- | --- | --- | --- | --- | --- | --- | --- | --- |
|  | **OR***^1^* | **95% CI***^2^* | **p-value** |  | **OR***^1^* | **95% CI***^2^* | **p-value** |  | **OR***^1^* | **95% CI***^2^* | **p-value** |
| Tobacco expenditure ^a^ |  |  |  |  |  |  |  |  |  |  |  |
| None | Ref. |  |  |  | Ref. |  |  |  | Ref. |  |  |
| 1 (Lowest) | 1.92 | 1.46, 2.51 | <0.001 |  | 1.52 | 1.19, 1.94 | <0.001 |  | 1.57 | 1.23, 2.01 | <0.001 |
| 2 | 2.49 | 1.94, 3.20 | <0.001 |  | 1.65 | 1.32, 2.06 | <0.001 |  | 1.68 | 1.34, 2.11 | <0.001 |
| 3 | 2.10 | 1.63, 2.71 | <0.001 |  | 1.48 | 1.18, 1.85 | <0.001 |  | 1.51 | 1.21, 1.90 | <0.001 |
| 4 | 2.73 | 2.14, 3.49 | <0.001 |  | 1.81 | 1.46, 2.24 | <0.001 |  | 1.85 | 1.49, 2.30 | <0.001 |
| 5 (Highest) | 2.65 | 2.07, 3.38 | <0.001 |  | 1.64 | 1.31, 2.04 | <0.001 |  | 1.69 | 1.36, 2.11 | <0.001 |
| Equiv. disposable income^b^ |  |  |  |  |  |  |  |  |  |  |  |
| 5 (Highest) | Ref. |  |  |  | Ref. |  |  |  | Ref. |  |  |
| 4 | 2.39 | 1.89, 3.00 | <0.001 |  | 1.89 | 1.51, 2.37 | <0.001 |  | 1.89 | 1.51, 2.36 | <0.001 |
| 3 | 4.35 | 3.49, 5.42 | <0.001 |  | 3.29 | 2.66, 4.08 | <0.001 |  | 3.26 | 2.63, 4.05 | <0.001 |
| 2 | 6.14 | 4.93, 7.65 | <0.001 |  | 3.80 | 3.06, 4.72 | <0.001 |  | 3.71 | 2.99, 4.62 | <0.001 |
| 1 (Lowest) | 9.14 | 7.35, 11.4 | <0.001 |  | 5.49 | 4.42, 6.82 | <0.001 |  | 5.32 | 4.27, 6.63 | <0.001 |
| Gender |  |  |  |  |  |  |  |  |  |  |  |
| Male | Ref. |  |  |  | Ref. |  |  |  | Ref. |  |  |
| Female | 1.37 | 1.22, 1.53 | <0.001 |  | 1.27 | 1.14, 1.42 | 0.003 |  | 1.28 | 1.14, 1.42 | 0.003 |
| Age |  |  |  |  |  |  |  |  |  |  |  |
| 15-34 | Ref. |  |  |  | Ref. |  |  |  | Ref. |  |  |
| 35-54 | 0.80 | 0.70, 0.92 | 0.001 |  | 0.98 | 0.86, 1.11 | 0.8 |  | 0.99 | 0.87, 1.12 | 0.8 |
| 55+ | 0.27 | 0.23, 0.31 | <0.001 |  | 0.65 | 0.56, 0.77 | <0.001 |  | 0.66 | 0.57, 0.78 | <0.001 |
| Liquidity |  |  |  |  |  |  |  |  |  |  |  |
| Low |  |  |  |  | Ref. |  |  |  | Ref. |  |  |
| Middle |  |  |  |  | 0.33 | 0.29, 0.38 | <0.001 |  | 0.33 | 0.29, 0.38 | <0.001 |
| High |  |  |  |  | 0.16 | 0.13, 0.19 | <0.001 |  | 0.16 | 0.14, 0.19 | <0.001 |
| Tenure |  |  |  |  |  |  |  |  |  |  |  |
| Owned outright |  |  |  |  | Ref. |  |  |  | Ref. |  |  |
| Mortgaged |  |  |  |  | 1.98 | 1.51, 2.06 | <0.001 |  | 1.99 | 1.51, 2.06 | <0.001 |
| Rented |  |  |  |  | 2.91 | 2.47, 3.38 | <0.001 |  | 2.89 | 2.46, 3.38 | <0.001 |
| Other |  |  |  |  | 2.61 | 1.43, 2.74 | <0.001 |  | 2.61 | 1.43, 2.73 | <0.001 |
| Household location |  |  |  |  |  |  |  |  |  |  |  |
| Greater capital city |  |  |  |  | Ref. |  |  |  | Ref. |  |  |
| Rest of state |  |  |  |  | 0.97 | 0.87, 1.08 | 0.6 |  | 0.98 | 0.88, 1.09 | 0.7 |
| Education |  |  |  |  |  |  |  |  |  |  |  |
| Degree or higher |  |  |  |  | Ref. |  |  |  | Ref. |  |  |
| Certificate to Diploma |  |  |  |  | 1.27 | 1.09, 1.47 | 0.001 |  | 1.27 | 1.10, 1.47 | 0.001 |
| No post-secondary qualifications |  |  |  |  | 1.19 | 1.03, 1.39 | 0.022 |  | 1.20 | 1.03, 1.39 | 0.019 |
| Lone parent |  |  |  |  |  |  |  |  |  |  |  |
| No |  |  |  |  | Ref. |  |  |  | Ref. |  |  |
| Yes |  |  |  |  | 1.55 | 1.33, 1.82 | <0.001 |  | 1.54 | 1.31, 1.80 | <0.001 |
| Gambling |  |  |  |  |  |  |  |  |  |  |  |
| No |  |  |  |  |  |  |  |  | Ref. |  |  |
| Yes |  |  |  |  |  |  |  |  | 0.85 | 0.75, 0.97 | 0.013 |
| Alcohol |  |  |  |  |  |  |  |  |  |  |  |
| Yes |  |  |  |  |  |  |  |  | Ref. |  |  |
| No |  |  |  |  |  |  |  |  | 1.05 | 0.94, 1.17 | 0.4 |
| *n* obs. | 10,036 |  |  |  | 10,036 |  |  |  | 10,036 |  |  |
| *Note:* ^1^CI = Confidence Interval; RR = Risk Ratio; ^a^ Tobacco expenditure levels = either zero expenditure or into five equally distributed household expenditure quintiles; ^b^ Disposable income = gross income minus income tax and levies, i.e. the net income available for consumption and saving ^#^ Gender, age and education are the characteristics of the household referenced person, who is a person chosen to represent a household in a survey | | | | | | | | | | | |

The following regression model uses logistic regression predicting a single financial stress indicator, going without meal(s) due to finances.

Table S3: Logistic regression models predicting going without meal(s)

|  | **Multivariable model (1)** | | |  | **Multivariable model (2)** | | |  | **Multivariable model (3)** | | |
| --- | --- | --- | --- | --- | --- | --- | --- | --- | --- | --- | --- |
|  | **OR***^1^* | **95% CI***^2^* | **p-value** |  | **OR***^1^* | **95% CI***^2^* | **p-value** |  | **OR***^1^* | **95% CI***^2^* | **p-value** |
| Tobacco expenditure^a^ |  |  |  |  |  |  |  |  |  |  |  |
| None | Ref. |  |  |  | Ref. |  |  |  | Ref. |  |  |
| 1 (Lowest) | 2.66 | 1.56, 4.53 | <0.001 |  | 2.07 | 1.19, 3.60 | 0.010 |  | 2.06 | 1.18, 3.61 | 0.011 |
| 2 | 2.59 | 1.58, 4.25 | <0.001 |  | 1.35 | 0.80, 2.28 | 0.3 |  | 1.34 | 0.79, 2.28 | 0.3 |
| 3 | 1.73 | 0.99, 3.02 | 0.056 |  | 1.08 | 0.61, 1.92 | 0.8 |  | 1.08 | 0.60, 1.92 | 0.8 |
| 4 | 3.05 | 1.97, 4.73 | <0.001 |  | 1.88 | 1.19, 2.97 | 0.007 |  | 1.87 | 1.18, 2.97 | 0.008 |
| 5 (Highest) | 4.11 | 2.73, 6.20 | <0.001 |  | 2.09 | 1.35, 3.25 | <0.001 |  | 2.09 | 1.34, 3.26 | 0.001 |
| Equiv. disposable income^b^ |  |  |  |  |  |  |  |  |  |  |  |
| 5 (Highest) | Ref. |  |  |  | Ref. |  |  |  | Ref. |  |  |
| 4 | 4.17 | 1.76, 9.90 | 0.001 |  | 2.93 | 1.23, 6.99 | 0.015 |  | 2.93 | 1.23, 6.99 | 0.015 |
| 3 | 6.12 | 2.65, 14.2 | <0.001 |  | 3.76 | 1.61, 8.79 | 0.002 |  | 3.77 | 1.61, 8.81 | 0.002 |
| 2 | 11.2 | 4.93, 25.4 | <0.001 |  | 5.13 | 2.22, 11.8 | <0.001 |  | 5.14 | 2.22, 11.9 | <0.001 |
| 1 (Lowest) | 23.8 | 10.6, 53.3 | <0.001 |  | 11.0 | 4.82, 25.1 | <0.001 |  | 11.1 | 4.81, 25.4 | <0.001 |
| Gender |  |  |  |  |  |  |  |  |  |  |  |
| Male | Ref. |  |  |  | Ref. |  |  |  | Ref. |  |  |
| Female | 1.35 | 1.05, 1.73 | 0.019 |  | 1.06 | 0.81, 1.40 | 0.7 |  | 1.06 | 0.81, 1.40 | 0.7 |
| Age |  |  |  |  |  |  |  |  |  |  |  |
| 15-34 | Ref. |  |  |  | Ref. |  |  |  | Ref. |  |  |
| 35-54 | 0.79 | 0.59, 1.07 | 0.13 |  | 1.13 | 0.83, 1.54 | 0.4 |  | 1.13 | 0.83, 1.54 | 0.5 |
| 55+ | 0.30 | 0.21, 0.42 | <0.001 |  | 1.01 | 0.69, 1.47 | >0.9 |  | 1.01 | 0.69, 1.47 | >0.9 |
| Liquidity |  |  |  |  |  |  |  |  |  |  |  |
| Low |  |  |  |  | Ref. |  |  |  | Ref. |  |  |
| Middle |  |  |  |  | 0.27 | 0.19, 0.40 | <0.001 |  | 0.27 | 0.19, 0.40 | <0.001 |
| High |  |  |  |  | 0.07 | 0.04, 0.16 | <0.001 |  | 0.07 | 0.04, 0.16 | <0.001 |
| Tenure |  |  |  |  |  |  |  |  |  |  |  |
| Owned outright |  |  |  |  | Ref. |  |  |  | Ref. |  |  |
| Mortgaged |  |  |  |  | 2.32 | 1.22, 4.41 | 0.011 |  | 2.32 | 1.22, 4.41 | 0.011 |
| Rented |  |  |  |  | 6.19 | 3.42, 11.2 | <0.001 |  | 6.20 | 3.43, 11.2 | <0.001 |
| Other |  |  |  |  | 0.42 | 0.05, 3.78 | 0.4 |  | 0.42 | 0.05, 3.79 | 0.4 |
| Household location |  |  |  |  |  |  |  |  |  |  |  |
| Greater capital city |  |  |  |  | Ref. |  |  |  | Ref. |  |  |
| Rest of state |  |  |  |  | 1.06 | 0.82, 1.37 | 0.7 |  | 1.06 | 0.81, 1.37 | 0.7 |
| Education |  |  |  |  |  |  |  |  |  |  |  |
| Degree or higher |  |  |  |  | Ref. |  |  |  | Ref. |  |  |
| Certificate to Diploma |  |  |  |  | 1.26 | 0.82, 1.94 | 0.3 |  | 1.26 | 0.82, 1.94 | 0.3 |
| No post-secondary qualifications |  |  |  |  | 1.59 | 1.04, 2.43 | 0.031 |  | 1.59 | 1.04, 2.43 | 0.031 |
| Lone parent |  |  |  |  |  |  |  |  |  |  |  |
| No |  |  |  |  | Ref. |  |  |  | Ref. |  |  |
| Yes |  |  |  |  | 1.86 | 1.33, 2.58 | <0.001 |  | 1.86 | 1.33, 2.59 | <0.001 |
| Gambling |  |  |  |  |  |  |  |  |  |  |  |
| No |  |  |  |  |  |  |  |  | Ref. |  |  |
| Yes |  |  |  |  |  |  |  |  | 1.00 | 0.72, 1.40 | >0.9 |
| Alcohol |  |  |  |  |  |  |  |  |  |  |  |
| Yes |  |  |  |  |  |  |  |  | Ref. |  |  |
| No |  |  |  |  |  |  |  |  | 0.99 | 0.74, 1.31 | >0.9 |
| *n* obs. | 10,036 |  |  |  | 10,036 |  |  |  | 10,036 |  |  |
| *Note:* ^1^CI = Confidence Interval; RR = Risk Ratio; ^a^ Tobacco expenditure levels = either zero expenditure or into five equally distributed household expenditure quintiles; ^b^ Disposable income = gross income minus income tax and levies, i.e. the net income available for consumption and saving ^#^ Gender, age and education are the characteristics of the household referenced person, who is a person chosen to represent a household in a survey | | | | | | | | | | | |

Many cases in the HES dataset contain imputed values. The following regression is identical to the regression the main article but excludes the 3,633 within-scope cases that had imputed values.

Table S4: Regression models predicting financial stress count, without imputation

|  | **Multivariable model (1)** | | |  | **Multivariable model (2)** | | |  | **Multivariable model (3)** | | |
| --- | --- | --- | --- | --- | --- | --- | --- | --- | --- | --- | --- |
|  | **RR***^1^* | **95% CI***^2^* | **p-value** |  | **RR***^1^* | **95% CI***^2^* | **p-value** |  | **RR***^1^* | **95% CI***^2^* | **p-value** |
| Tobacco expenditure ^a^ |  |  |  |  |  |  |  |  |  |  |  |
| None | Ref. |  |  |  | Ref. |  |  |  | Ref. |  |  |
| 1 (Lowest) | 1.92 | 1.46, 2.53 | <0.001 |  | 1.52 | 1.20, 1.94 | <0.001 |  | 1.57 | 1.23, 1.99 | <0.001 |
| 2 | 2.45 | 1.89, 3.17 | <0.001 |  | 1.65 | 1.32, 2.05 | <0.001 |  | 1.67 | 1.34, 2.08 | <0.001 |
| 3 | 2.20 | 1.73, 2.81 | <0.001 |  | 1.60 | 1.30, 1.97 | <0.001 |  | 1.65 | 1.34, 2.03 | <0.001 |
| 4 | 2.37 | 1.88, 3.00 | <0.001 |  | 1.53 | 1.25, 1.86 | <0.001 |  | 1.57 | 1.29, 1.92 | <0.001 |
| 5 (Highest) | 2.29 | 1.83, 2.87 | <0.001 |  | 1.51 | 1.24, 1.83 | <0.001 |  | 1.55 | 1.28, 1.88 | <0.001 |
| Equiv. disposable income e^b^ |  |  |  |  |  |  |  |  |  |  |  |
| 5 (Highest) | Ref. |  |  |  | Ref. |  |  |  | Ref. |  |  |
| 4 | 2.06 | 1.66, 2.54 | <0.001 |  | 1.64 | 1.34, 2.00 | <0.001 |  | 1.62 | 1.32, 1.98 | <0.001 |
| 3 | 2.88 | 2.35, 3.53 | <0.001 |  | 2.15 | 1.77, 2.62 | <0.001 |  | 2.12 | 1.74, 2.57 | <0.001 |
| 2 | 4.64 | 3.81, 5.66 | <0.001 |  | 2.87 | 2.37, 3.47 | <0.001 |  | 2.76 | 2.28, 3.35 | <0.001 |
| 1 (Lowest) | 7.59 | 6.25, 9.21 | <0.001 |  | 4.41 | 3.65, 5.33 | <0.001 |  | 4.19 | 3.45, 5.08 | <0.001 |
| Gender |  |  |  |  |  |  |  |  |  |  |  |
| Male | Ref. |  |  |  | Ref. |  |  |  | Ref. |  |  |
| Female | 1.24 | 1.12, 1.37 | <0.001 |  | 1.11 | 1.01, 1.22 | 0.036 |  | 1.11 | 1.01, 1.22 | 0.038 |
| Age |  |  |  |  |  |  |  |  |  |  |  |
| 15-34 | Ref. |  |  |  | Ref. |  |  |  | Ref. |  |  |
| 35-54 | 0.85 | 0.75, 0.97 | 0.016 |  | 1.02 | 0.91, 1.15 | 0.7 |  | 1.03 | 0.92, 1.16 | 0.6 |
| 55+ | 0.37 | 0.32, 0.42 | <0.001 |  | 0.79 | 0.68, 0.91 | 0.001 |  | 0.80 | 0.69, 0.92 | 0.002 |
| Liquidity |  |  |  |  |  |  |  |  |  |  |  |
| Low |  |  |  |  | Ref. |  |  |  | Ref. |  |  |
| Middle |  |  |  |  | 0.35 | 0.31, 0.39 | <0.001 |  | 0.35 | 0.31, 0.40 | <0.001 |
| High |  |  |  |  | 0.26 | 0.23, 0.30 | <0.001 |  | 0.26 | 0.23, 0.30 | <0.001 |
| Tenure |  |  |  |  |  |  |  |  |  |  |  |
| Owned outright |  |  |  |  | Ref. |  |  |  | Ref. |  |  |
| Mortgaged |  |  |  |  | 1.52 | 1.29, 1.78 | <0.001 |  | 1.52 | 1.29, 1.79 | <0.001 |
| Rented |  |  |  |  | 2.22 | 1.92, 2.58 | <0.001 |  | 2.21 | 1.90, 2.56 | <0.001 |
| Other |  |  |  |  | 2.23 | 1.67, 2.97 | <0.001 |  | 2.20 | 1.65, 2.94 | <0.001 |
| Household location |  |  |  |  |  |  |  |  |  |  |  |
| Greater capital city |  |  |  |  | Ref. |  |  |  | Ref. |  |  |
| Rest of state |  |  |  |  | 0.91 | 0.82, 1.00 | 0.044 |  | 0.91 | 0.83, 1.00 | 0.054 |
| Education |  |  |  |  |  |  |  |  |  |  |  |
| Degree or higher |  |  |  |  | Ref. |  |  |  | Ref. |  |  |
| Certificate to Diploma |  |  |  |  | 1.19 | 1.04, 1.36 | 0.010 |  | 1.19 | 1.05, 1.36 | 0.008 |
| No post-secondary qualifications |  |  |  |  | 1.27 | 1.11, 1.45 | <0.001 |  | 1.27 | 1.11, 1.45 | <0.001 |
| Lone parent |  |  |  |  |  |  |  |  |  |  |  |
| No |  |  |  |  | Ref. |  |  |  | Ref. |  |  |
| Yes |  |  |  |  | 1.60 | 1.39, 1.84 | <0.001 |  | 1.58 | 1.37, 1.81 | <0.001 |
| Gambling |  |  |  |  |  |  |  |  |  |  |  |
| No |  |  |  |  |  |  |  |  | Ref. |  |  |
| Yes |  |  |  |  |  |  |  |  | 0.91 | 0.81, 1.02 | 0.10 |
| Alcohol |  |  |  |  |  |  |  |  |  |  |  |
| Yes |  |  |  |  |  |  |  |  | Ref. |  |  |
| No |  |  |  |  |  |  |  |  | 1.09 | 0.99, 1.21 | 0.082 |
| *n* obs. | 6,403 |  |  |  | 6,403 |  |  |  | 6,403 |  |  |
| *Note:* ^1^CI = Confidence Interval; RR = Risk Ratio; ^a^ Tobacco expenditure levels = either zero expenditure or into five equally distributed household expenditure quintiles; ^b^ Disposable income = gross income minus income tax and levies, i.e. the net income available for consumption and saving ^#^ Gender, age and education are the characteristics of the household referenced person, who is a person chosen to represent a household in a survey | | | | | | | | | | | |


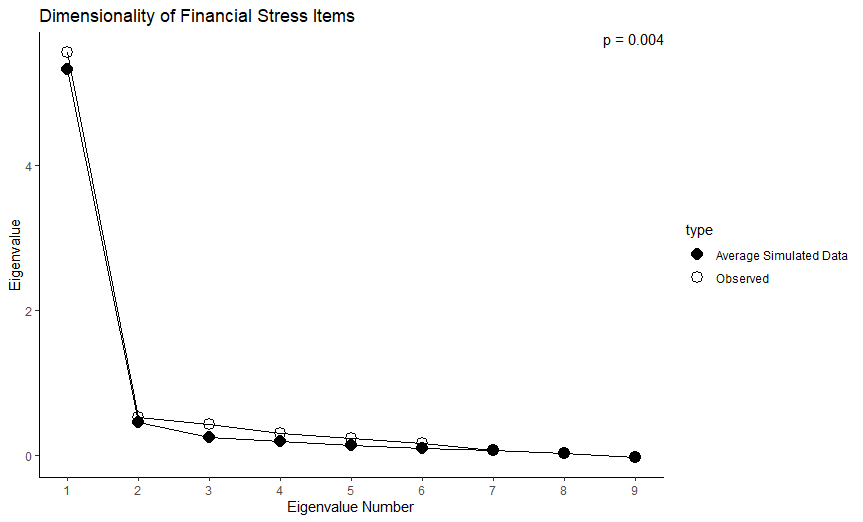


Figure S1. The scree plot of dimensionality of financial stress items

## Additional information on study variables

### Household reference person definition

To determine the household reference person, these increasingly selective criteria were applied to all household members aged 15 years or older until only one person remained.

- the person with the highest tenure when ranked as follows: owner without a mortgage, owner with a mortgage, renter, other tenure
- one of the partners in a registered or de facto marriage, with dependent children
- one of the partners in a registered or de facto marriage, without dependent children
- a lone parent with dependent children
- the person with the highest income
- the eldest person.

### Financial stress variable

Here we detail the questionnaire items used to collect the financial stress indicators used in our analyses. Three items in the questionnaire provided us with data on nine indicators of financial stress, this was because the first item was a multiple-answer question with seven possible responses, while the other two responses each provided data on a single indicator of financial stress.

The full questionnaire is available from the Australian Bureau of Statistics at catalogue number 6503.0. The following information is copyright of the Commonwealth of Australia 2017 and available under creative commons licence (BY-NC-ND 3.0 AU).

#### Multiple-answer item

The first questionnaire item was a single multiple-answer question:

Over the past year, have any of the following happened to [you/your household] because of a shortage of money?

Respondents were allowed to select as many options as applied to them out of the following:

| Option | Prompt |
| --- | --- |
| 1 | Could not pay for electricity, gas or telephone bills on time |
| 2 | Could not pay for car registration or insurance on time |
| 3 | Pawned or sold something |
| 4 | Went without meals |
| 5 | Unable to heat my home |
| 6 | Sought assistance from welfare/community organisations |
| 7 | Sought financial help from friends or family |

####

#### Multiple-choice items

The other two questionnaire items we used as financial stress indicators were the availability of emergency money and subjective financial situation.

**Emergency money**

If all of a sudden [you / your household] had to get two thousand dollars for something important, could the money be obtained within a week?

Which had ‘*yes*’ or ‘*no*’ as the possible responses. We coded ‘*no*’ as being indicative of financial stress.

**Financial situation**

*Thinking of [your / your household’s] situation over the last 12 months, which one of the following statements best describes [your/your household’s] financial situation?*

Which had the options ‘*Spend more money than we get*’, ‘*Just break even most weeks*’, or ‘*Able to save money most weeks*’. We coded ‘*Spend more money than we get*’ as being indicative of financial stress.
